# Simplified Post-stroke Functioning Assessment Based on ICF *via* Dichotomous Mokken Scale Analysis and Rasch Modeling

**DOI:** 10.3389/fneur.2022.827247

**Published:** 2022-04-14

**Authors:** Chun Feng, Zhong-Li Jiang, Ming-Xue Sun, Feng Lin

**Affiliations:** ^1^The First Rehabilitation Hospital of Shanghai, Rehabilitation Hospital Affiliated to Tongji University, Shanghai, China; ^2^Sir Run Run Hospital, Nanjing Medical University, Nanjing, China; ^3^Maternal and Child Health Care Hospital of Jiangyin, Jiangyin, China

**Keywords:** Mokken scale analysis, Rasch modeling, item response theory, International Classification of Functioning, Disability and Health (ICF), ICF core set, stroke

## Abstract

**Purpose:**

This study aims to accomplish two tasks for *International Classification of Functioning, Disability and Health* (ICF) application among persons with stroke: (1) to make an ICF tool for measuring personal abilities with simplified assessment operations; (2) to quantitatively evaluate ICF categories for being functioning rather than being disabled.

**Methods:**

A total of 130 inpatients with stroke *via* convenience sampling were evaluated by the extended comprehensive ICF core set for stroke, modified Rankin scale, and modified Barthel index (MBI). This study investigated the responses to 118 stroke-related ICF items (59 items in b and d domains individually) using Mokken scale analysis followed with Rasch modeling.

**Results:**

A Mokken scale with 47 items was extracted from the binary data (1 as no-impairment or mild-impairment and 0 as moderate to complete impairment). A Rasch model with 45 items was derived from the Mokken scale. The conversion chart was available involving the original ordinal scores to Rasch-transformed scores from 0 to 100 (interval scale). Total scores exhibited a high correlation with the personal abilities estimated by the Rasch model. The personal ability also demonstrated a significantly strong correlation with the score of the MBI. Thus, the 45 ICF items were suggested to rate potential functional ability as a single measurement.

**Conclusion:**

Based on simple “functioning or disabled” judgment tasks, ICF assessment can be simplified to a questionnaire with answering “yes-or-no” questions for each category. Functioning level for each person and difficulty of being functioning for each category can be estimated by the Rasch model of this questionnaire.

## Introduction

At present, available functional assessments in the clinical practice include the Barthel Index (BI) for basic activities of daily-living evaluation without considering cognition status, which also contains a floor and ceiling effect for assessing activity and participation (1). On the other hand, the 36-Item Short-Form Health Survey (SF-36) involving 8 scales is the most extensively quality-of-life assessment. However, its developers point out that the total score of SF-36 cannot be a single measure for quality of life (2). The widely used scale such as the National Institutes of Health stroke scale was originally designed for acute care settings but might not be appropriate for rehabilitation practices (3).

Moreover, at present, certain assessment scales are mostly developed for specific diseases. It causes difficulty to obtain assessment with a universal standard for patients with multiple diseases (such as metabolic syndrome). Patients with stroke often suffer from comorbidities such as hypertension or diabetes (4). Patients might also experience a variety of functional disorders during the continuum of the diseases. Even patients with good recovery as assessed by traditional ways can still retain cognitive, emotional, or social integration impairments (5). Therefore, a sufficiently comprehensive assessment with high efficiency is required for the status determination of patients' activity and participation.

In 2001, the *International Classification of Functioning, Disability and Health* (ICF) as a theoretical framework and classification system was promulgated by WHO. ICF is designed to describe human experience regarding health under the umbrella terms of functioning and disability (6). The ICF qualifier scale is a 5-point Likert scale with numeric rating ranks “no impairment” = 0; “mild impairment” = 1, “moderate impairment” =2, “severe impairment” = 3, and “complete impairment” = 4. There are several controversies regarding how to carry out such assessments (7, 8). (1) Consensuses are not achieved regarding the rating standard, especially in the determination of rating 2–4. (2) The vast number of ICF items for each disease and each item requires to rate from qualifier 0–4, which is too overwhelming to apply into clinical evaluation. (3) The core sets cannot provide a single measurement for the individual's functional level simply by the sum of scores. (4) The categories are not estimated by their difficulty or easiness of being functioning or being disabled. All categories are treated as non-hierarchic items in the core sets. The former two problems are about the efficiency of assessment. The latter two limit the sufficiency of assessment. The efficiency means that the task is time-saving. The sufficiency implies that the result is comprehensive with details. The currently available ICF core sets incline to the sufficiency rather than efficiency. It is a challenge to make a simplified comprehensive assessment tool based on the comprehensive core set.

To measure personal ability and to quantitatively differentiate item difficulties, scholars introduced the item response theory (IRT) and utilized its parametric method of Rasch modeling in several ICF studies, namely, the brief ICF core set (9, 10), rehabilitation set (11), spinal cord injury core set (12, 13), and Lucerne ICF-based multidisciplinary observation scale (14). However, these models did not solve the problem of efficiency. The 5-point qualifier system remained unchanged. In recent years, non-parametric IRT models based on Mokken scale analysis (MSA) have begun to attract attention in the medical areas (15–17). In comparison with the necessary Guttman hypothesis held by the Rasch model, which means that highly competent subjects must be bound to score on the easy tasks (18), MSA holds the probabilistic Guttman hypothesis that high-ability subjects were more likely to complete low-difficulty tasks. Several conditions, namely, single scale, local independence, monotonicity, and item invariant item ordering (IIO) can be feasibly checked by MSA. These are conditions required for further parametric IRT processes, especially Rasch modeling (19). That is, the MSA offers preparation processes of data shaping and hypothesis testing for Rasch modeling.

The purpose of this multicenter, cross-sectional study was to provide an ICF-based dichotomous-scoring scale and its relative Rasch model to assess personal ability and item difficulty among the Chinese stroke population. In comparison with the five-point scoring ICF system, the dichotomous-scoring scale was simplified. We assumed that the final scale with high reliability and validity was based on the MSA and Rasch modeling. The simplified ICF scale might be a promising tool for evaluating individual functional levels. The final Rasch model offered an estimation of difficulty being functional for each item.

## Methods

### Subject Recruitment and Study Design

This study is a multicentral and cross-sectional study from October of 2018 to June of 2020 involving 130 inpatients' acute, subacute, or chronic phases of recovery from stroke by convenience sampling. To increase the result generalizability for convenience sampling, we followed a maximum variation sampling strategy to capture the common patterns from a great deal of heterogeneity (20).

After providing written informed consent, participants were asked to complete a one-h interview and clinical examination in a private room. The demographic information collected included gender, age, marital status, and education level. The diagnostic category, recurrence, plegia side, body mass index, and the duration of disease were reviewed through medical charts or face-to-face interviews. Based on the interview and clinical examination, two trained evaluators were administered to evaluate modified Rankin scale (MRS) and Modified Barthel Index (MBI) and rate 0–4, 8 (no specified), or 9 (not applicable) for the extended comprehensive ICF core set for stroke questionnaire (21). The ICF categories scored 8 and 9 were defined as items with missing data. Persons with missing ratios > 30% were excluded because there was no reliably unbiased missing value imputation method (22). The eligibility criteria included (21): (1) diagnosis of stroke by the computed tomography (CT) or magnetic resonance imaging (MRI); (2) stroke with plegia as the main diagnosis; (3) age ≥ 18 years old; and (4) able to provide the informed consent. The exclusion criteria: (1) unhealed trauma or surgical incision; (2) patients with critical illness, such as cardiopulmonary failure; and (3) other diseases affecting data collection, such as a history of mental illness or severe dementia.

Extended comprehensive ICF core set for stroke consists of 166 items (21), namely, 59 items related to body function (b), 59 items to activity and participation (d), 37 items to environmental and individual factors (e), and 11 items to body structure (s). This study sorted out the b and d categories (Appendix 1) and excluded e and s categories. The main reason was that this study was completed in the inpatient setting. There was the same tendency of e categories for the patients admitted to the hospital. For instance, items regarding the information of health practitioners, relatives, and families tended to attain similar scores in the conditions of hospitalization. In addition, the qualifier of e categories is scored differently than b, d, and s. The s categories with non-interventional items, such as the structure of the brain, were also excluded.

This study was approved by the ethics committee of two local rehabilitation hospitals in Shanghai and Nanjing and informed consent was obtained from all subjects before the experiment.

### Data Analysis

**Figure 1:**
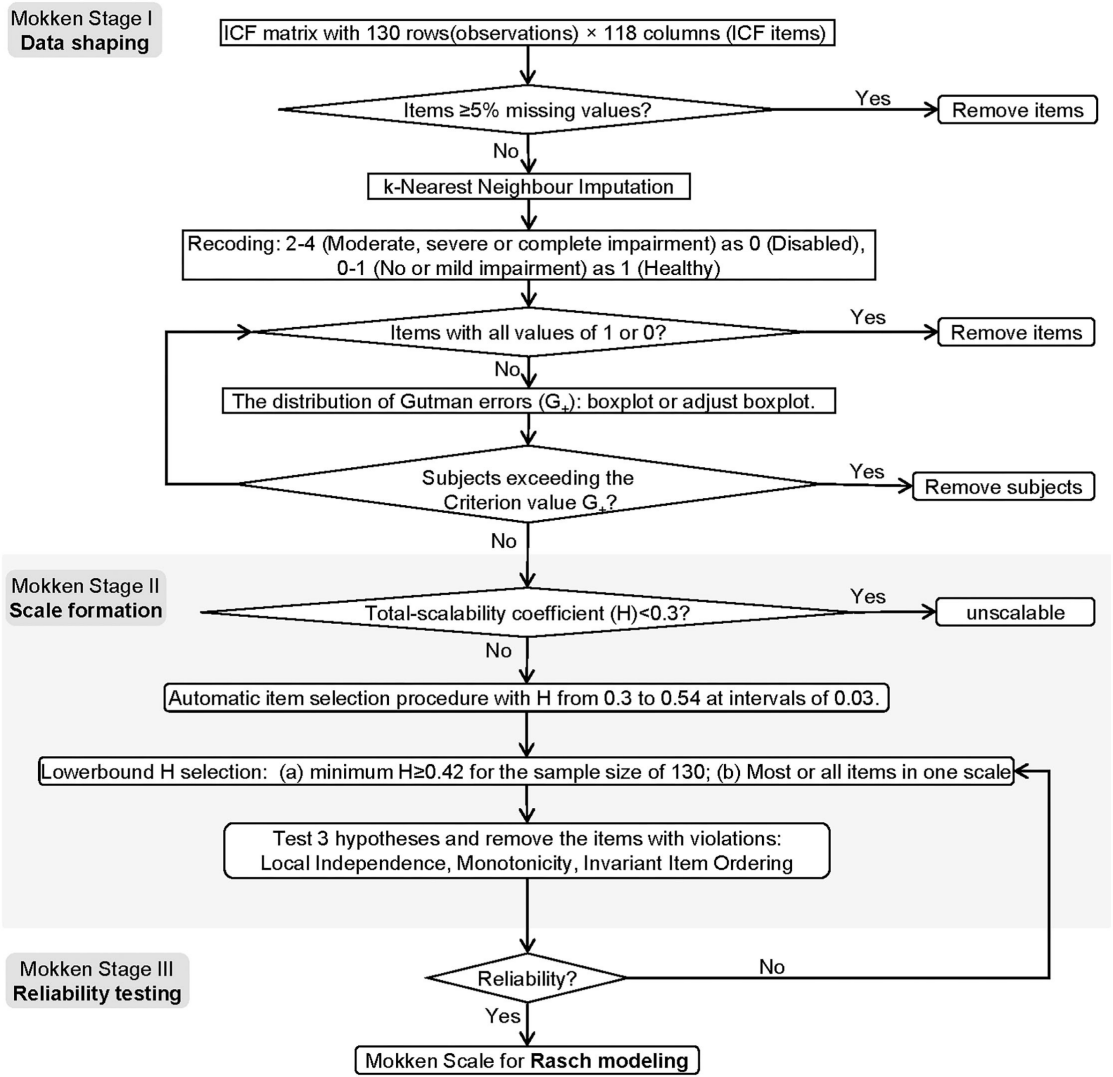
The flowchart of the MSA. The flowchart contains three stages, namely, date shaping, scale formation, and reliability testing.

**Figure 2:**
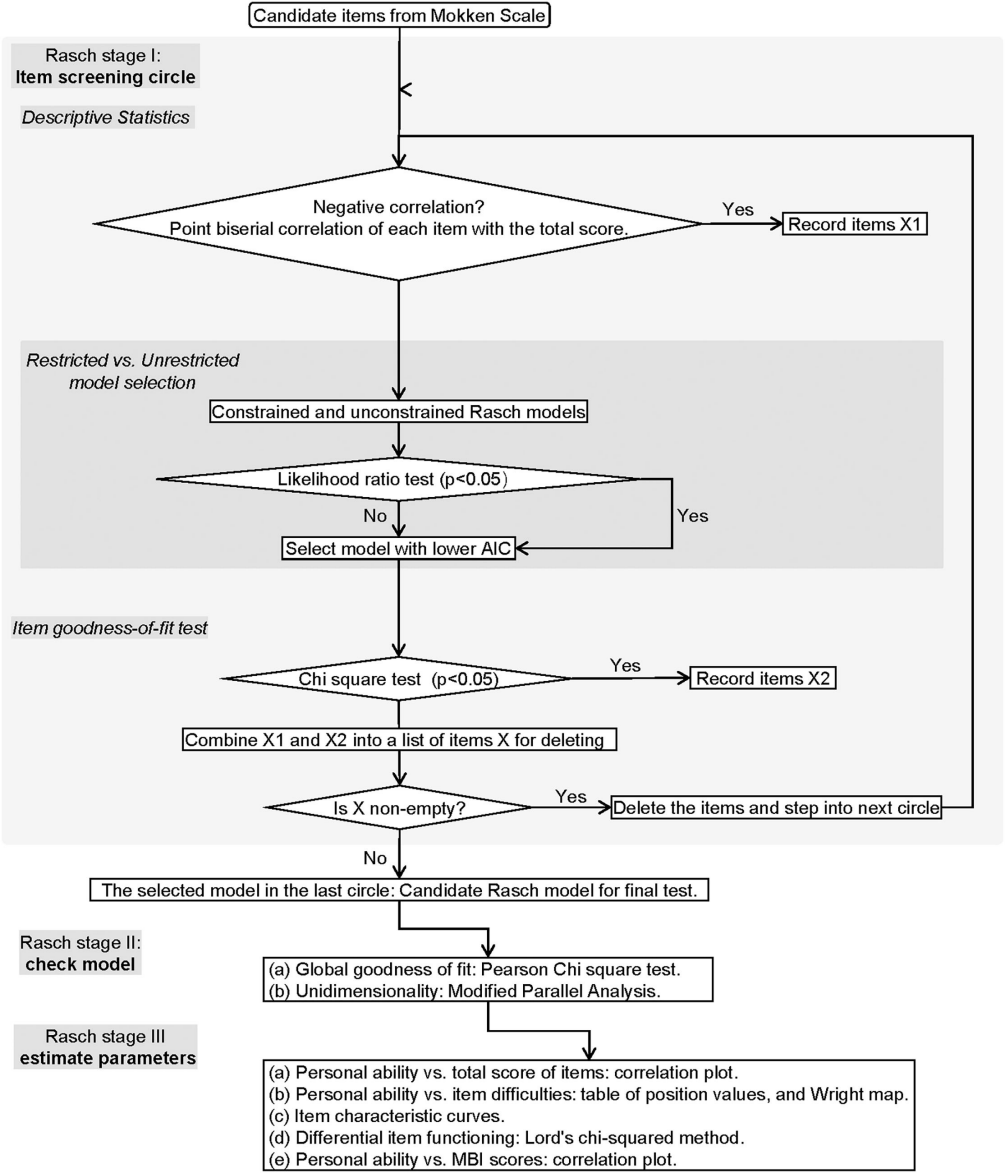
The flowchart of Rasch modeling. The flowchart demonstrates the process of Rasch modeling including item screening, model identification, and parameter estimation.

The brief flowchart of MSA and the Rasch modeling were exemplified in Figures 1, 2. The MSA utilized the “mokken” package of R and the guideline of Sijtsma and van der Ark (23). The Rasch modeling was with the “ltm” package of R (24). The study method was reviewed by an expert in the IRT field.

#### MSA Stages

##### MSA I: Data Shaping

To shrink imputation size, we applied a relatively conservative cut value (item missing values ≥ 5%) to exclude the categories. The censored data were completed by *k*-nearest neighbor imputation with *k* = 5. The imputed data were then binarized using the following criteria: no or mild impairment as 1 (functioning), and moderate, severe, or complete impairment as 0 (disabled). We excluded the participants who were outliers in the distribution of the number of Guttman's errors (G_+_) (22). Constantly valued items should be removed for controlling ceiling or floor effects.

##### MSA II: Scale Formation

First, the global scalability coefficient (denoted as *H*) of the items was calculated. It denotes the discrimination power of the items (25). According to Sijtsma and van der Ark (23), if the *H* < 0.3, the set is unscalable; if 0.3 ≤ *H* < 0.4, the set is a weak scale; if 0.4 ≤ *H* < 0.5, it is a medium scale; if *H* ≥ 0.5, the scale is strong.

Second, the automatic item selection procedure (AISP) was applied based on the genetic algorithms (26). This genetic parameter includes the size of sampling items = 20, the cross-over probability = 0.05, and mutation probability = 0.1. The scalability coefficient boundary value starts from 0.3 to 0.54 (step length = 0.03). To meet the minimum sample size requirements for MSA (27), the threshold value was selected 0.42.

*Hypothesis I: Local Independence*. The heat map was based on a pairwise scalability coefficient between items i and j (denoted as H_ij_). The monotone homogeneity model of the Mokken scale implies that 0 ≤ H_ij_ ≤ 1. The values outside this range are violations. W values were calculated based on conditional association to estimate the degree of a certain item that is suspected regarding the local dependence (28). The extreme values of W (denoted as W_+_) are identified by the Tukey fence algorithm: W_+_ > Q3+3^*^(Q3-M). M is the median and Q3 is the 3rd percentile. The minimum sample size of a rest-score group was 4. No weight was set for the sample size on each conditional covariance. The weight of each conditional covariance on the computation of W1, W2, and W3 was the proportion of negative covariances. The minimum sample size of the conditioning variable to compute a covariance was 4.

*Hypothesis II: Monotonicity*. The parameter has set the minimum sample size as 50 and the lowest item response function value as 0.03 (15). Four indices will be reported. (1) #ac: the number of possible violations; (2) #vi: the actual number of violations; (3) #zsig: number of statistically significant violations, 0 indicating no violations; and (4) Crit: the critical value summarizes effect size of violation (29). Although Crişan et al. (29) reported that the Cirt has poor power given a small sample size of 100, we decided to report this routine index for more comprehensive considerations.

*Hypothesis III: IIO*. i.e., The items have a fixed rank of difficulty irrespective of the level of personal ability. The function was set as *manifest invariant item ordering (MIIO)* by conducting a backward item selection procedure to make the final decision.

The items violating one of the three hypotheses were removed. The remained items entered the reliability testing step.

##### MSA III: Reliability Testing

Four reliability statistics were calculated: Cronbach's α, Guttman's λ_2_, Molenaar Sijtsma ρ, and the latent class reliability coefficient (LCRC) (25). They all range from 0 to 1. A larger value indicates stronger internal consistency. The Cronbach's α and the Guttman's λ_2_ are two traditional coefficients of reliability. However, they may generate biased estimations for non-parametric models. The Molenaar Sijtsma ρ is more suitable for scales with IIO. The LCRC provides unbiased estimations for the Mokken scale without limiting the condition of IIO.

#### Rasch Modeling Stages

##### Rasch Stage I: Item Screening Circle

First, point biserial correlation with a negative coefficient between each item and the total score (either the total score with or without the certain item score) was all examined. The flagged items were recorded in the list of X1.

Second, an optimal model is selected from the constrained (discrimination = 1) and unconstrained (discrimination ≠ 1) Rasch models based on the likelihood ratio test. The model with the lower value of the Akaike information criterion (AIC) is preferred.

Third, the item goodness-of-fit test was performed based on χ^2^ statistics. The items with *p* < 0.05 were recorded in the list of X2.

Fourth, if the combination of X1 and X2 is non-empty, the items in it will be removed from the candidate set and the next circle will start. If the combination set is empty, the circle will stop.

##### Rasch Stage II: Model Checking

*Global Goodness-of-Fit Testing*. The global goodness-of-fit of the model was tested by the parametric bootstrap test using Pearson's χ^2^ statistic. The null hypothesis states that the observed data have been generated under the Rasch model with parameter values of the maximum likelihood estimates $\hat{\theta }$. The specific test method is to simulate the standard Rasch model based on the estimated parameter $\hat{\theta }$ from the candidate Rasch model. As a result, it produces simulated data sets (number = *B*), each of which can calculate the Pearson χ^2^ value (denoted as *T*_*b*_). The observed data can also calculate the Pearson χ^2^ value (denoted as *T*_*obs*_). By calculating the number of *T*_*b*_ > *T*_*obs*_ (denoted as *N*_+_), the *p*-value can be achieved by the following equation: (1+*N*_+_)/(*B*+1). If *p* > 0.05, it supports *H*_0_, i.e., the measured values are determined with confidence from the simulated standard Rasch model based on the same parameters of the candidate Rasch model. We set the *B* = 199 for this procedure.

*Unidimensional Testing*. A total of 100 unidimensional models were built with the Monte Carlo simulation method. The alternative hypothesis was that the second eigenvalue of the observed data is substantially larger than the second eigenvalue of data under the assumed model. If the test shows *p* > 0.05, it indicates the candidate model is not significantly different from the simulated unidimensional models.

If the candidate model passes both tests, it becomes the final Rasch model for further parameter estimating.

##### Rasch Stage III: Parameters Estimating

*Personal Ability vs. Total Score of Items*. The correlation was assessed between the total score of the items and the normalized personal ability level estimated by the final Rasch model.

*Item Positions*. The item difficulties, i.e., the item positions, were estimated for the final Rasch model. The χ^2^ test was exploited to check the goodness of fit for each item in the model. The Bonferroni method was used to calibrate the *p*-value. Items with adjusted *p* > 0.05 fit good to the model.

*Item Characteristic Curve (ICC)*. Visualization for checking the shape and relation of curves, especially monotonicity and IIO.

*Differential Item Functioning Analysis*. In this study, value 1 stood for “functioning or no disabled” concerning the ICF items. Lord's χ^2^ analysis embedded in the “difR” package of R was employed to analyze the gender as the only DIF of the model (30). The *p*-values of multiple comparisons were adjusted by Holm adjustment.

*The Estimated Personal Ability vs. the MBI*. Pearson correlation coefficient was estimated for justification of using the Rasch model to measure functioning levels.

## Results

### Demographics

A total of 130 stroke patients with pelgia (36 women and 94 men) were recruited in this research. As described in mean ± SD [minimum, maximum], the age was 64.9 ± 13 [28, 87] years. The duration of stroke was 4.46 ± 8.62 [0, 58] months, the MBI was 47.7 ± 25.7 [0, 100] scores, and the body mass index was 24.3 ± 3.85 [15.62, 39.86] kg/m^2^. There was no significant difference in education time (*p* = 0.324), solitary status (*p* = 0.774), diagnosis (*p* = 0.475), recurrence (*p* = 1.000), plegia side (*p* = 0.286), age (*p* = 0.1213), the course of disease (*p* = 0.1629), body mass index (*p* = 0.6305), and scores of MRS (*p* = 0.675) and MBI (*p* = 0.6305) across gender. The detailed description of the demographic data was manifested in Table 1.

**Table 1:** Description of demographic data and characteristics of the disease [mean (SD) or number (percent)].

**Factor**	**Level**	**Both**	**Female**	**Male**	***p***
Gender		130 (100%)	36 (27.7%)	94 (72.3%)	
Education time	<6 years	12 (9.23%)	6 (50.0%)	6 (50.0%)	0.324
	6–9 years	48 (36.9%)	13 (27.1%)	35 (72.9%)	
	10–12 years	33 (25.4%)	9 (27.3%)	24 (72.7%)	
	>12 years	37 (28.5%)	8 (21.6%)	29 (78.4%)	
Solitary	No	105 (80.8%)	28 (26.7%)	77 (73.3%)	0.774
	Yes	25 (19.2%)	8 (32.0%)	17 (68.0%)	
Diagnosis	Hemorrhage	34 (26.2%)	12 (35.3%)	22 (64.7%)	0.475
	Infarction	95 (73.1%)	24 (25.3%)	71 (74.7%)	
	Both	1 (0.77%)	0 (0.00%)	1 (100%)	
Recurrence	No	93 (71.5%)	26 (28.0%)	67 (72.0%)	1.000
	Yes	37 (28.5%)	10 (27.0%)	27 (73.0%)	
Plegia side	Left	60 (46.2%)	20 (33.3%)	13 (86.7%)	0.286
	Right	55 (42.3%)	14 (25.5%)	40 (66.7%)	
	Both	15 (11.5%)	2 (13.3%)	41 (74.5%)	
Modified rankin scale	1	11 (8.46%)	1 (9.09%)	10 (90.9%)	0.675
	2	19 (14.6%)	6 (31.6%)	13 (68.4%)	
	3	21 (16.2%)	5 (23.8%)	16 (76.2%)	
	4	65 (50.0%)	20 (30.8%)	45 (69.2%)	
	5	14 (10.8%)	4 (28.6%)	10 (71.4%)	
Post-stroke stage	≤ 2 weeks	17 (13.1%)	2 (5.56%)	15 (16.0%)	0.212
	3–27 weeks	94 (72.3%)	30 (83.3%)	64 (68.1%)	
	≥28 weeks	19 (14.6%)	4 (11.1%)	15 (16.0%)	
Age (years)		64.9 (13.0)	67.89 (14.00)	63.70 (12.43)	0.1213
Course of disease (months)		4.46 (8.62)	3.22 (4.12)	4.94 (9.79)	0.1629
Modified Barthel Index (scores)		47.7 (25.7)	45.97 (24.69)	48.35 (26.20)	0.6305
Body mass index (kg/m^2^)		24.3 (3.85)	23.92 (4.43)	24.45 (3.61)	0.5213

*Gender differences were estimated by χ^2^ test for categorical data and t-test for continuous data*.

#### MSA Results

##### MSA I: Data Shaping

**Figure 3:**
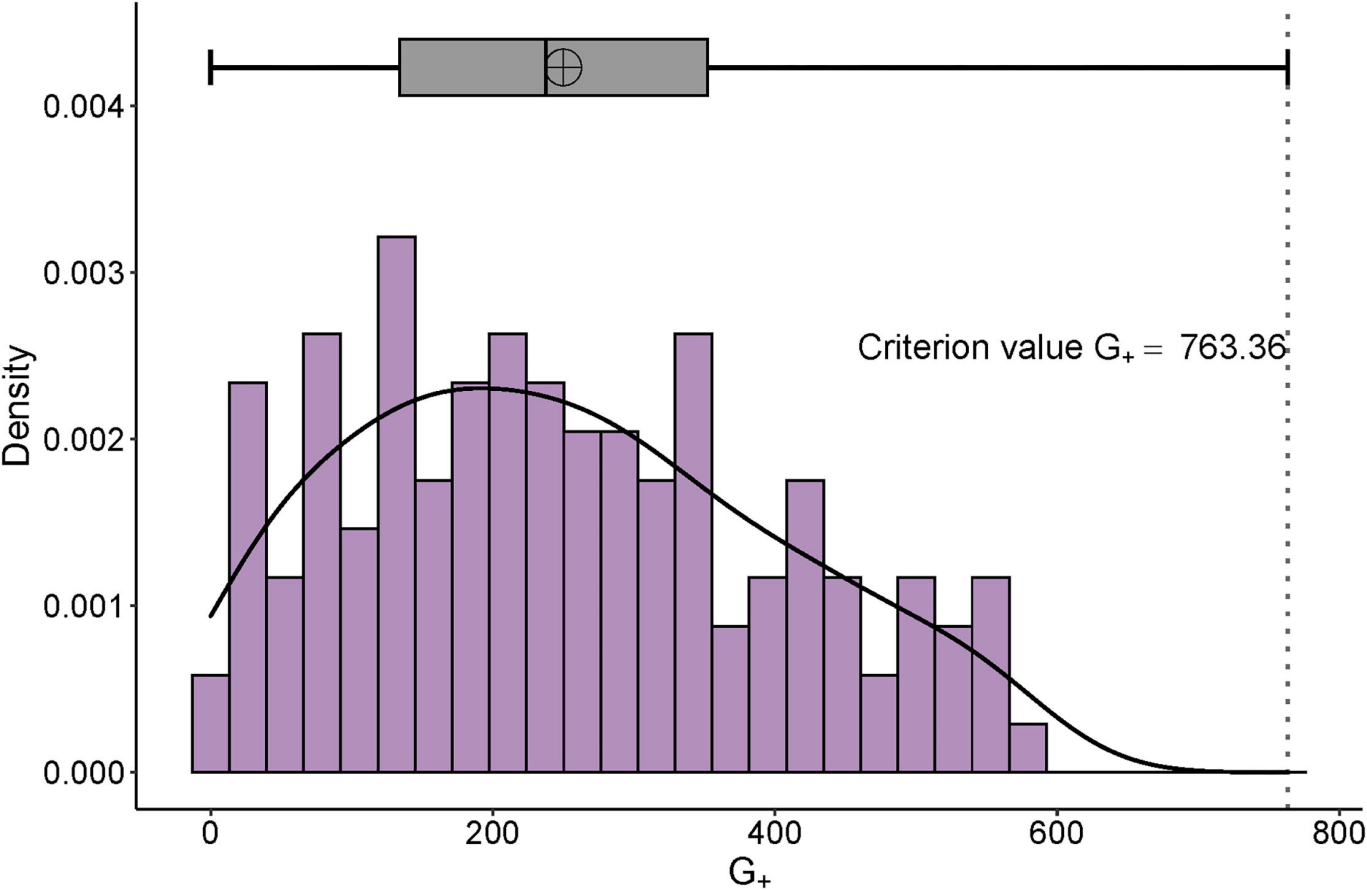
Distribution of G_+_ and its adjusted boxplot. The right-skewed distribution of personal G_+_ can be seen. The x-axis is the number of the G_+_ and the y-axis represents the probability density.

All participants were below the extreme value and qualified for further analysis (Figure 3, adjusted criterion value G_+_ = 763.36). According to the screening criterion of ≥ 5% censored data, there were 25 items removed. Appendix 1 reveals 57 items of b, and 36 items of d category were further analyzed by MSA.

##### MSA II: Scale Formation

*Global Scalability Coefficient Estimating*. The H of the 93 items = 0.3619 (standard error = 0.0401), which was less accuracy (0.3 < H < 0.4) for generating the Mokken scale and ought to be improved by further item selection procedures (23).

*AISP*. Appendix 2 listed the outcome patterns of AISP with different cut values of scalability coefficients. Sample size in the range of 50–250 requires at least a cut value of *c* = 0.42(27) to retain the predominance of scale 1 and construct a unidimensional model (Appendix 3).

*Homogeneity Coefficients Measuring*. Appendix 4 demonstrated that the overall homogeneity coefficient was 0.5446 with a standard error of 0.0437. Two things should be noted: according to the standard errors of “b110 Consciousness functions,” “b117 Intellectual functions,” “b180 Experience of self and time functions,” “b430 Hematological system functions,” “b450 Additional respiratory functions,” and “b540 General metabolic functions” more than 0.1, these 6 items demonstrate the relatively low accuracy of the measurement. However, the H_i_ of 50 items is all > 0.42, which suggests that the sample size is suitable for the MSA of this item set.

**Figure 4:**
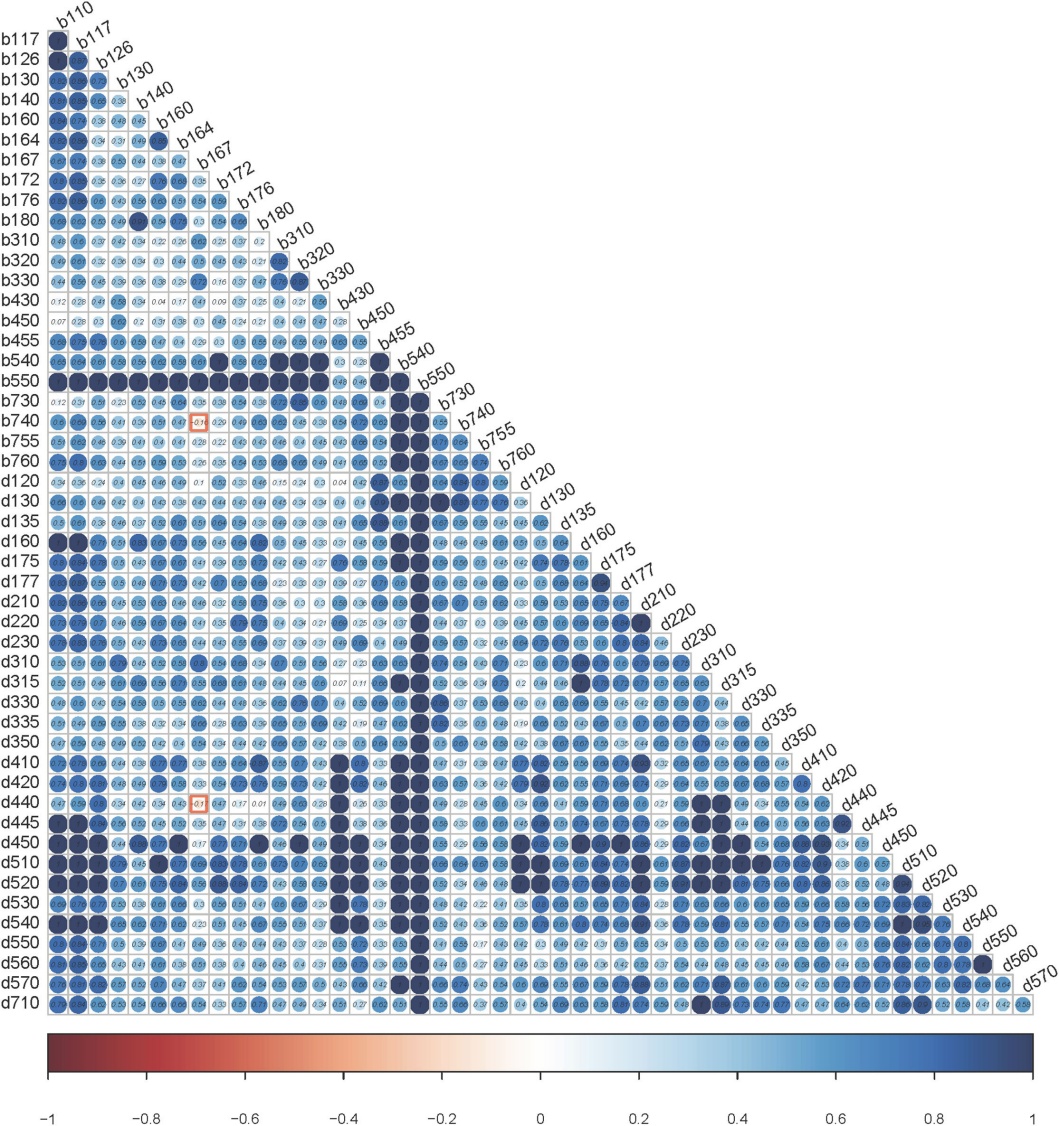
Heatmap using scalability coefficients for pair of items. The values of Hij are within the dots. The bar scale underneath is from red (value = −1) to blue (value = 1). The color of the dots can reflect the degree of violation. The bluer it is, the less likely it is to violate the local dependency.

*Hypothesis I: Local Independence*. The scalability coefficient Hij is the normed covariance between items i and j. Negative values violate the hypotheses of the monotone homogeneity model of the Mokken scale, and positive values tend to but do not necessarily support the monotonicity, local independence, and unidimensionality. Visualization of H_ij_ (Figure 4) revealed only two violations with H_ij_ <0 included “b167 Mental functions of language” with “b740 Muscle endurance functions” (H_ij_ = −0.16), and b167 with “d440 Fine hand use” (H_ij_ = −0.17). Under the test of W values based on conditional association, items met the local independence hypothesis.

*Hypothesis II: Monotonicity*. There are no violations of monotonicity (Appendix 5).

*Hypothesis III: IIO* (Appendix 6). Although there were 6 items (b167, b330, b755, d130, d160, and d570) with significant violations, four of them (b167, b330, b755, and d160) with Crit values > 40. Finally, the backward item selection procedure revealed that 47 items reached the criteria of MIIO except for the “b167 Mental functions of language,” “b330 Fluency and rhythm of speech functions,” and “b755 Involuntary movement reaction functions.”

##### MSA III: Reliability Testing

The reliability testing confirmed that the scale included the remained 47 items with high reliability: Cronbach's α = 0.9533, Guttman's λ_2_ = 0.9566, Molenaar Sijtsma ρ = 0.9622, and LCRC = 0.9731.

#### Rasch Modeling Results

##### Rasch Stage I: Item Screening Circle

The loop was completed after 2 runs of the circle with two items (“b172 calculation functions” and “d440 fine hand use”) violating the item goodness of fit. The constrained Rasch model comprising 45 items moved on to the next stage.

##### Rasch Stage II: Model Checking

The global goodness-of-fit test was met (*p* =0.335). The unidimensional test revealed that the measured second eigenvalue was 3.79, while the average second eigenvalue of the Monte Carlo simulation model was 5.17 (*p* = 0.8614). Thus, there was no significant difference between the measured model and the simulated unidimensional model.

##### Rasch Stage III: Parameters Estimating

**Figure 5:**
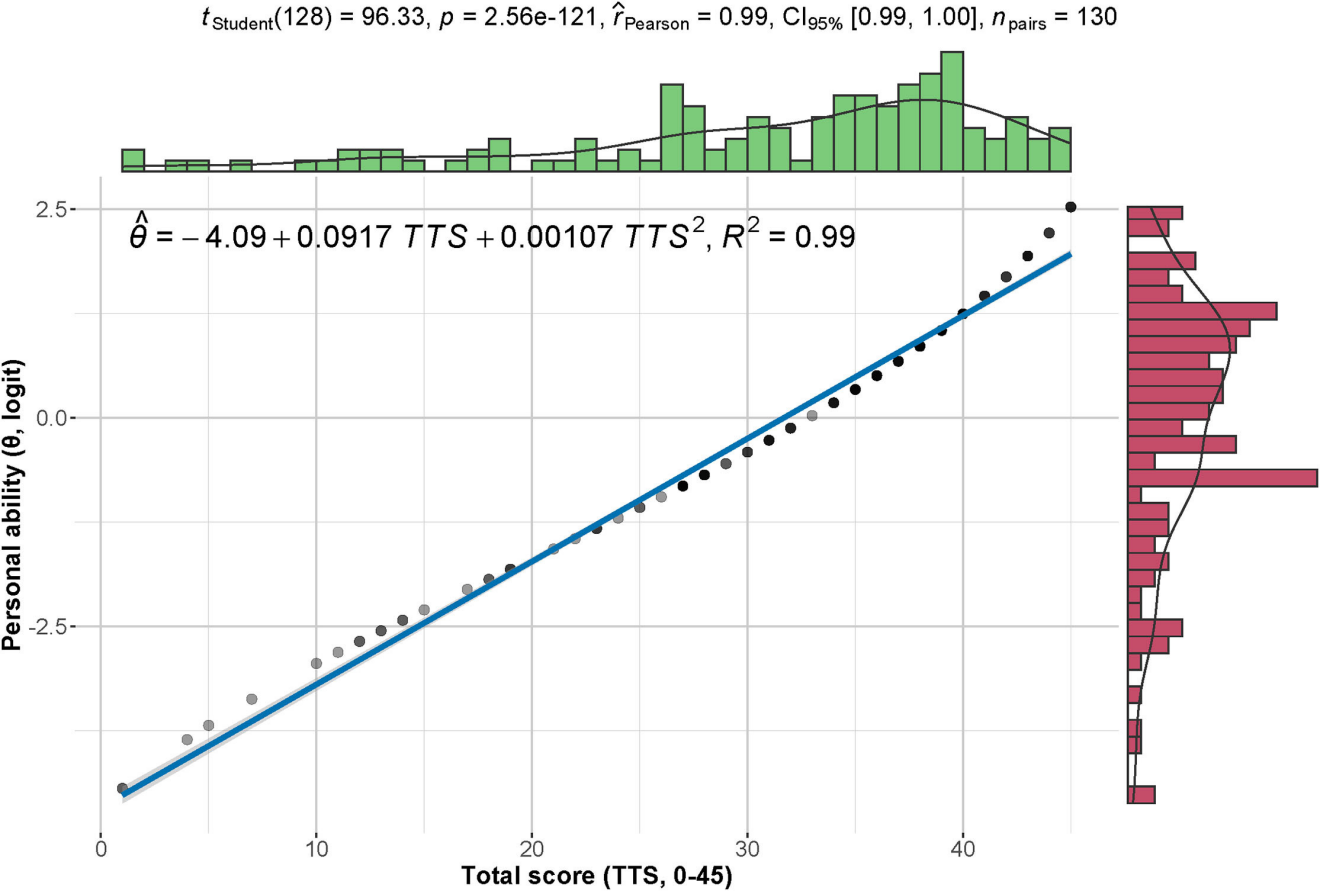
Correlation between total scores and personal abilities. The total scores (0–45) demonstrate a strong positive correlation with the normalized personal abilities (logit). The density distribution of each index is shown on the corresponding margin.

The Pearson correlation between the total scores and normalized personal abilities was significantly strong (Figure 5, *p* = 2.56 × 10^−121^ < 0.001, effect size = 0.99). The equation for the estimated value of personal ability is $\hat{\theta }=\;-\;4.09\;+\;0.0917TTS\times 0.00107TTS^{2}$ ($\hat{\theta }$ represents the logit value; TTS stands for total score of the assessment scale). The 45 items generated a scale that can estimate personal competence by using the sum of its face values in form of binary scores. For improving the goodness of fit, we applied a quadratic model for the linear relationship between the total score and the percentage value of normalized personal ability.

**Figure 6:**
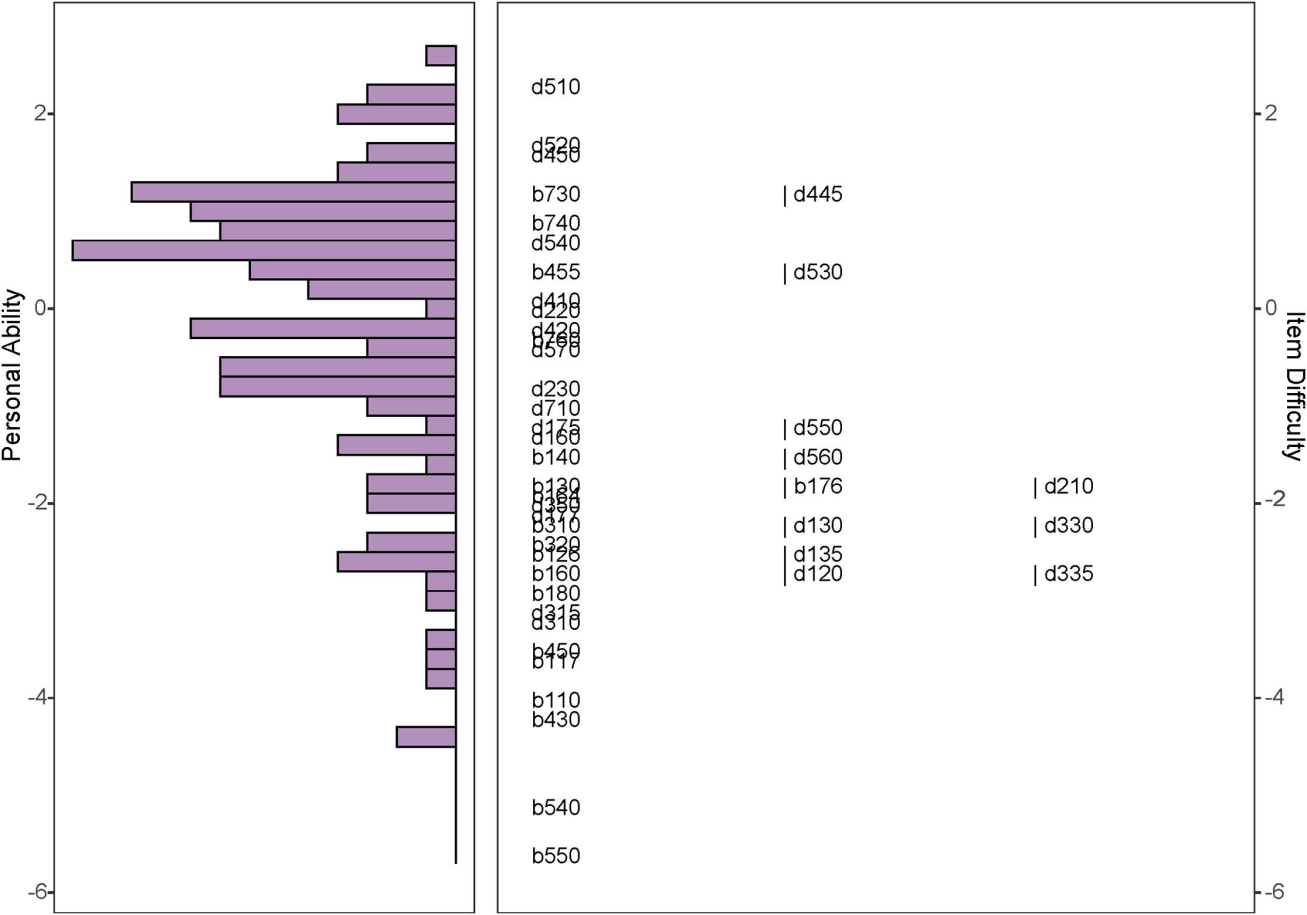
Wright map of the selected Rasch model. The Wright map shows the distributions of personal ability and item difficulty on a uniformed logit scale. The right panel manifests the item difficulty. The higher position of the items is, the more difficult items are. The left panel exhibits personal ability. The length of each column indicates the number of people at the same ability level.

Figure 6 is the Wright map of the final Rasch model that showed the distributions of personal ability and item difficulty among those ICF categories. The negative peak of personal ability revealed that more participants had a relatively high ability. Appendix 7 and the right panel of Figure 6 suggest that four items were relatively easy, namely, “b110 Consciousness functions,” “b430 Hematological system functions,” “b540 General metabolic functions,” and “b550 Thermoregulatory functions.”

**Figure 7:**
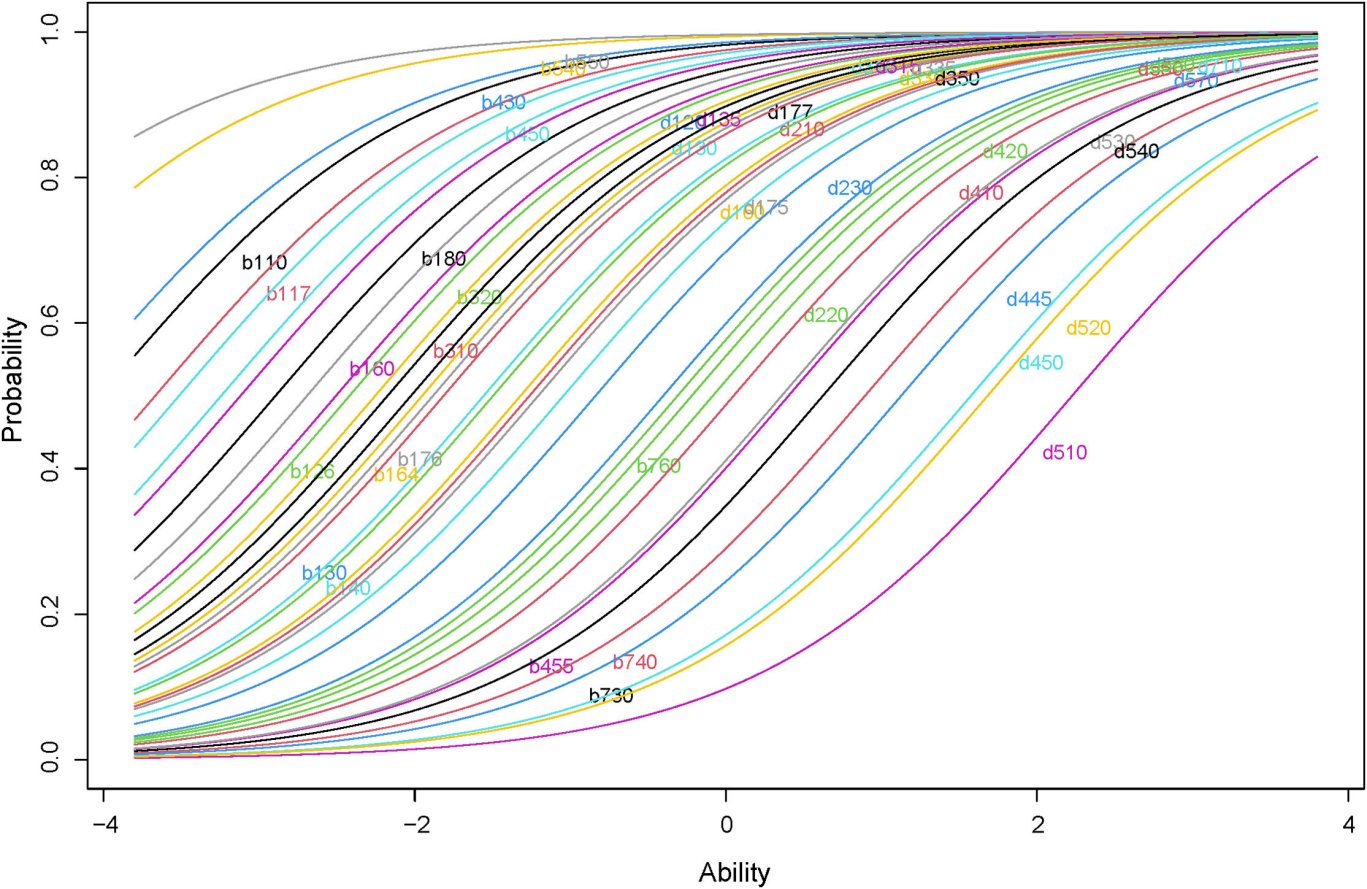
ICC of the selected Rasch model. The plot displays the ICC of the final Rash model. The curves are ranked in a paralleled pattern and are shaped with monotonically increasing.

Figure 7 is the ICC of the final Rash model. The “S” shapes and parallel distributions of the curves supported monotonicity and IIO.

**Figure 8:**
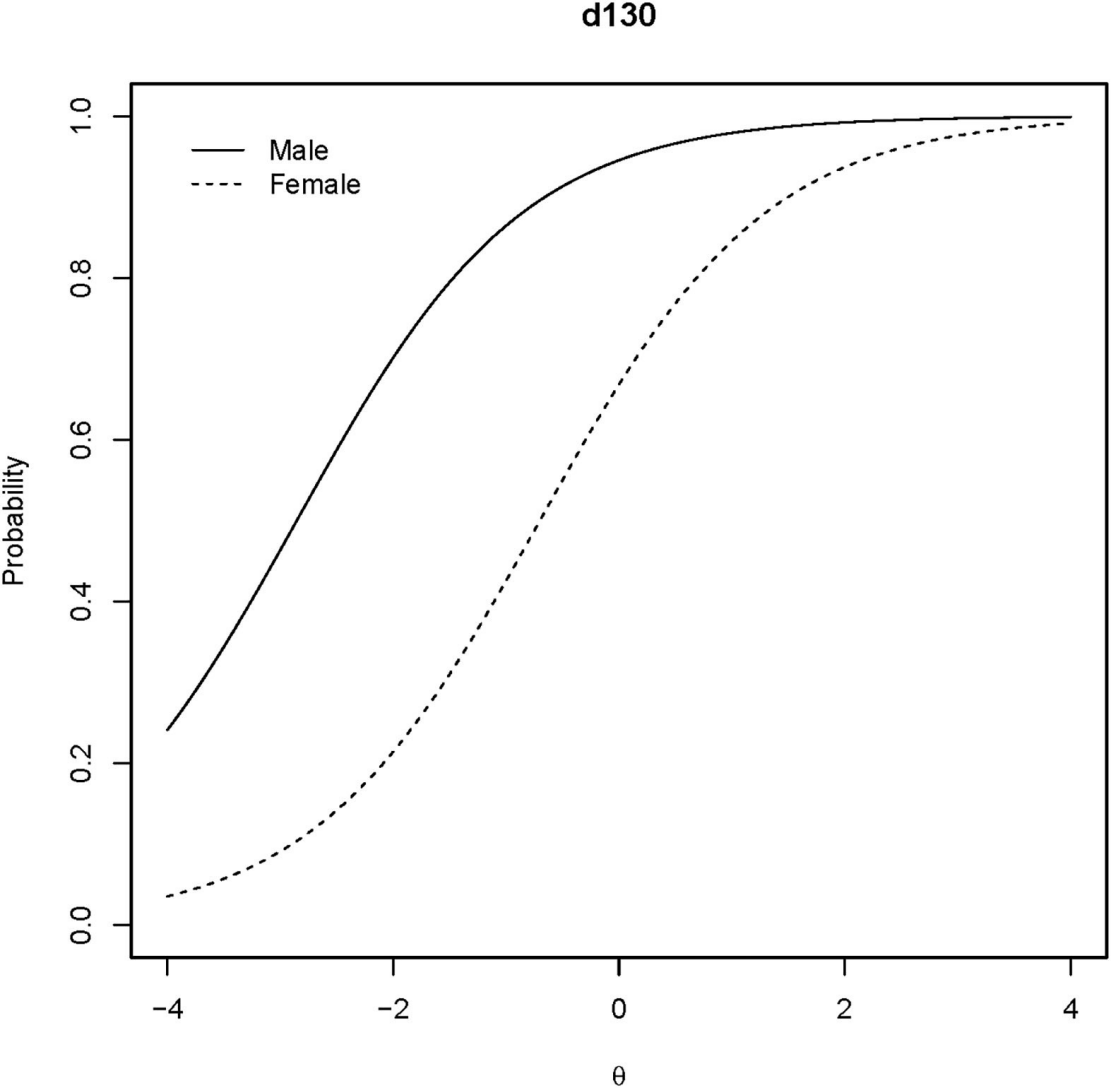
ICCs of the DIF item. The ICCs for the DIF of the “d130” item are plotted regarding gender.

Appendix 8 shows Lord's χ^2^ test for differential item functioning analysis of gender. The Holm adjusted *p*-values suggested that the items are not DIF items of gender except for the “d130 Copying” (Holm adjusted *p* = 0.0261 < 0.05). Figure 8 shows that the “d130” is a uniformed DIF. The Welch two-sample *t*-test for personal abilities considering genders showed *t*_(64.59)_ = −0.3432 with *p* = 0.7325. The absolute difference in means of personal abilities is 0.1021 logit.

**Figure 9:**
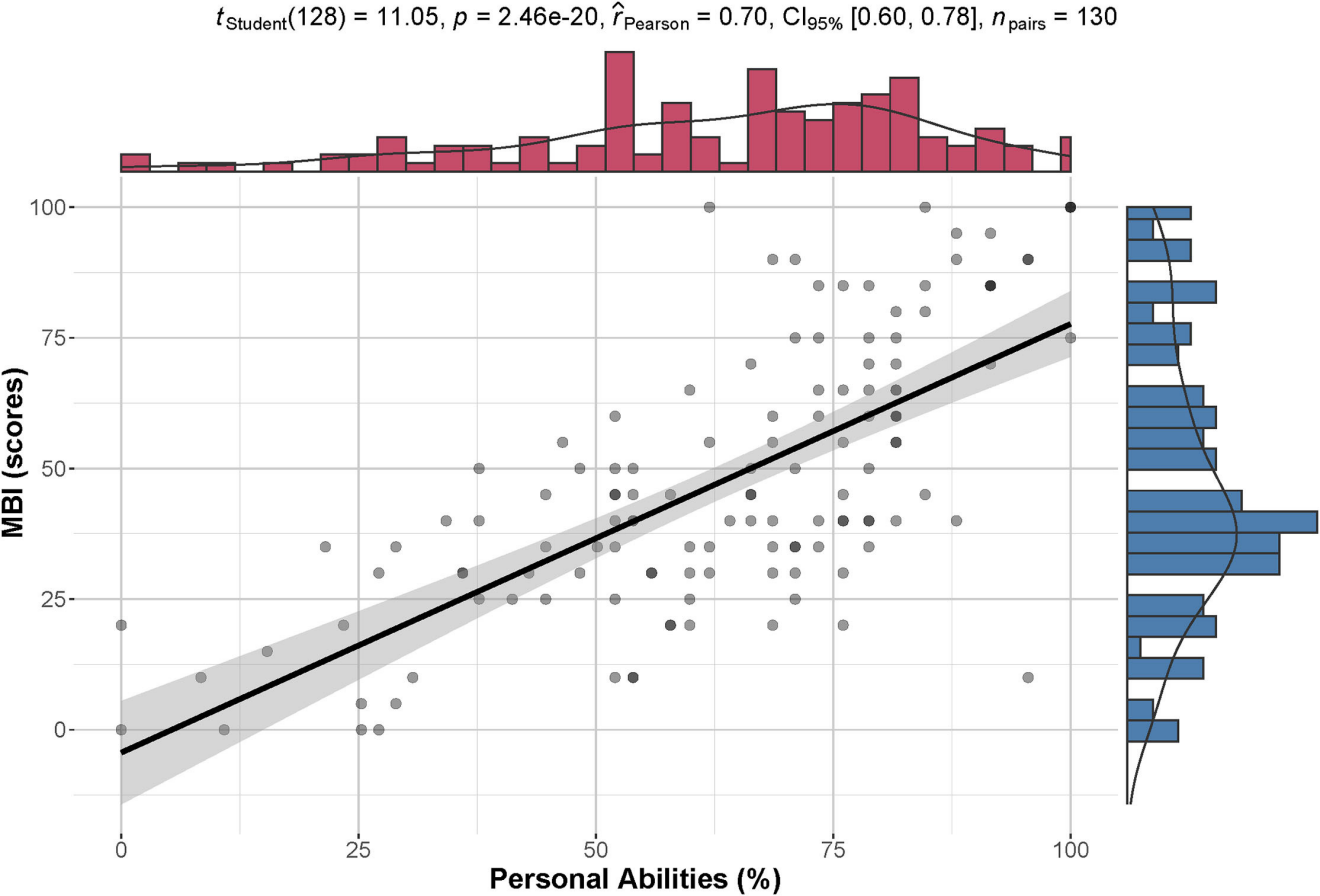
Correlation between personal ability and MBI. There is a high correlation between standardized personal ability (%) and MBI (scores). The density distribution of each index is shown on the corresponding margin.

Figure 9 shows the Pearson correlation between individual ability and MBI score. It suggested that the personal abilities calculated in the model had a strong correlation with MBI (*p* = 2.46 × 10^−20^ < 0.001 and effect size = 0.70).

Table 2 is the final assessment scale containing 45 items. The conversional table between the total scores of 45-ICF items and functional ability in percentile score (0–100%) can be found in Table 3.

**Table 2:** The final assessment scale with the Rasch model.

**Categories**	**Category title**	**Disabled (0)**	**Functioning (1)**
b110	Consciousness functions		
b117	Intellectual functions		
b126	Temperament and personality functions		
b130	Energy and drive functions		
b140	Attention functions		
b160	Thought functions		
b164	Higher-level cognitive functions		
b176	Mental function of sequencing complex movements		
b180	Experience of self and time functions		
b310	Voice functions		
b320	Articulation functions		
b430	Hematological system functions		
b450	Additional respiratory functions		
b455	Exercise tolerance functions		
b540	General metabolic functions		
b550	Thermoregulatory functions		
b730	Muscle power functions		
b740	Muscle endurance functions		
b760	Control of voluntary movement functions		
d120	Other purposeful sensing		
d130	Copying		
d135	Rehearsing		
d160	Focusing attention		
d175	Solving problems		
d177	Making decisions		
d210	Undertaking a single task		
d220	Undertaking multiple tasks		
d230	Carrying out daily routine		
d310	Communicating with–receiving–spoken messages		
d315	Communicating with–receiving–non-verbal messages		
d330	Speaking		
d335	Producing non-verbal messages		
d350	Conversation		
d410	Changing basic body position		
d420	Transferring oneself		
d445	Hand and arm use		
d450	Walking		
d510	Washing oneself		
d520	Caring for body parts		
d530	Toileting		
d540	Dressing		
d550	Eating		
d560	Drinking		
d570	Looking after one's health		
d710	Basic interpersonal interactions		

*The condition of the disabled refers to moderate, severe, or complete dysfunction. The condition healthy refers to no or mild dysfunction*.

**Table 3:** The converted form between 45-ICF item scores and functional ability (0–100%).

**Total score**	**θ (Logit)**	**Percent**	**Total score**	**θ (Logit)**	**Percent**
0	−4.0889	0	23	−1.4149	43
1	−3.9961	1	24	−1.2731	45
2	−3.9011	3	25	−1.1291	47
3	−3.8041	5	26	−0.983	49
4	−3.7049	6	27	−0.8348	52
5	−3.6035	8	28	−0.6844	54
6	−3.5001	9	29	−0.5319	57
7	−3.3945	11	30	−0.3773	59
8	−3.2867	13	31	−0.2205	62
9	−3.1769	15	32	−0.0616	64
10	−3.0649	16	33	0.0994	67
11	−2.9507	18	34	0.2625	69
12	−2.8345	20	35	0.4278	72
13	−2.7161	22	36	0.5952	75
14	−2.5956	24	37	0.7648	77
15	−2.4729	26	38	0.9365	80
16	−2.3481	28	39	1.1103	83
17	−2.2212	30	40	1.2862	85
18	−2.0922	32	41	1.4643	88
19	−1.961	34	42	1.6445	91
20	−1.8277	36	43	1.8269	94
21	−1.6922	38	44	2.0113	97
22	−1.5546	40	45	2.198	100

*Total score (TTS) represents the scores of 45-ICF items. The theta (Logit) can be calculated by the equation $\hat{\theta }$ = −4.09 + 0.0917TTS × 0.00107TTS^2^. Percent is the normalized personal ability in percentile score (0–100%)*.

## Discussion

This study provides research examples (Supplementary Materials 1, 2) and application ideas for health assessment based on the IRT. By following the pipeline of MSA and Rasch modeling, it was realized by the simple ICF questionnaire (0 = disabled and 1 = functioning) to complete competence evaluation. The total score of the Mokken scale and the estimated value of the personal functional ability of the Rasch model represented the measure of functioning degree.

Rasch modeling has long been used as an ICF study approach. It was an ideal theoretical model for estimating ordered questionnaires (9, 11, 12, 14) for transforming the ordinal scale into the linear interval scale. In addition, both personal ability and item difficulty can be calculated in a Rasch model (14). MSA was performed as the preliminary screening of the scale content. It can not only reduce the complicated data processing by Rasch but also increase the stability and universality of the scale (23). Rusch et al. (31) utilized MSA to extract scalable items that are compatible with the hypotheses for further parametric modeling. We followed this strategy by applying the MSA filtering before the Rasch modeling that constructs the potential functional assessment scale.

The content of our final scale is similar to the subscale of motor, communication, and cognition in Van de Winckel et al. (14). However, there are differences in the item selection between the Lucerne ICF-based multidisciplinary observation scale (LIMOS) and our scale. The LIMOS only contains activities and participation-related ICF items, while the scale we develop not only involves activity and participation (d) but also body function (b) based on the extended ICF core set for stroke. Like the LIMOS, a higher functional level is with a higher score and represents less disabled. However, the final scale in our study can also be easily answered by “Yes” or “No” without considering the 5-point Likert scale with numeric rating ranks “no impairment” = 0; “mild impairment” = 1, “moderate impairment” =2, “severe impairment” = 3, and “complete impairment” = 4.

Our scale has fewer items (45 categories) than the initial b and d items (118 categories) in the extended comprehensive stroke ICF core set but contains extensive items compared to the brief stroke core set (18 categories) (32). Since the yes-or-no response is the simplest task of the questionnaire, our dichotomous model makes it possible to easily embed the scale in clinical practices. It can relieve health practitioners from the burden of multiple assessment scales.

The significantly strong correlations between personal ability and MBI indicated that the scale can measure the activities of daily living. If we dig into the details of the final scale, most of the 10 aspects of MBI were covered by the categories, especially the items with bold fonts in Appendix 7. The final 45-item scale included more aspects of daily functioning, for example, “d570 Looking after one's health,” “d710 Basic interpersonal interactions,” and “b130 Energy and drive functions.” Moreover, the model not only provided an assessment tool but also offered meaningful insights for intervention. For instance, “d510 Washing oneself,” “d520 Caring for body parts,” and “d450 Walking” are the items with top difficulties. These are essential activities for health experiences and are usually scheduled as main rehabilitation aims.

We should emphasize that the application of the final scale assigns 1 for “functioning” and 0 for “disability.” Rasch model offers two critical values, namely, personal ability and item difficulty. If the disability score = 1, the personal ability means “the disability level of the person,” and the item difficulty implies “the difficulty of the item to be dysfunction.” If the functioning score = 1, the personal ability signifies “the functional level of the person,” and the item difficulty indicates “the difficulty of the item to be healthy.” Previous reports emphasize the purpose of estimating the functional level for a person rather than focusing on clinical intervention for a specific ICF item. Therefore, they score functioning as 0 and disability as 1. They did not differentiate the “difficulty of being dysfunction” and the “difficulty of being healthy.” In contrast, if the functioning is scored as 1 and disability as 0, it can provide the physicians and therapists with item difficulty values that are more in line with their intuitive clinical thinking. It also provides information on whether the certain ICF item should be one of the rehabilitation targets (Is the function deficit difficult to address) or where its rank (How hard the ICF item is) should be put on the intervention schedule of several targets.

The sample size is one of the limitations of this study. Although we controlled this limitation by referring to the study of Straat et al. (27) and followed the principle of maximum variation sampling strategy (20), the ideal solution is still to be large sampling. In addition, our study participants were Chinese people. Considering the cultural differences, our study results might capture the characteristics of stroke among the Chinese population and might not be suitable for other cultural backgrounds. The third limitation is the validity of Table 3, although we provided evidence of the correlation between personal ability and MBI, improvements in the future study could include more powerful and widely accepted tools such as the SF-36, functional independence measure, and Fugl–Meyer scale. The fourth limitation is described in our inclusion criterion. All the post-stroke persons were diagnosed with plegia and capable of providing consent. Therefore, regarding the rank of item difficulties, some higher brain functioning categories are relatively easy, such as “d350 Conversation,” “d177 Making decisions,” “b126 Temperament and personality functions,” and “b117 Intellectual functions.” Following the workflow of this study, the model can be furtherly expanded among the post-stroke population without plegia. The fifth aspect that can be improved is other parametric models. Except for difficulty and discrimination as 1, the final scale can be deeply analyzed by multiple parameters such as pseudoguessing and careless responding. By using multidimensional models, more items with scale number 2, 3, or 4 may be included. In this study, only the gender DIF was explored based on our sample size. The DIF values will be further predicted by education time, solitary status, age (below and above 60 years old), diagnostic category, and the duration of disease after recruiting more participants.

However, the primary aim of this study is to provide a simple ICF-based tool for assessing functioning levels in persons with stroke. The resulted Mokken scale and Rasch model are not perfect, but they provide the first step to improve this strategy.

## Conclusion

This study dealt with two questions about the ICF application. First, we evaluated the degree of health (functioning) itself rather than focusing on certain dysfunctional conditions (disability). Second, we completed the quantitative assessment of personal abilities for Chinese stroke persons diagnosed with plegia. This study put forward new ideas of calculating individual functioning through the MSA-based Rasch model. The 45-item scale generated from MSA and Rasch analysis can be an assessment tool for potential functional competence. Moreover, the final scale can guide the grading of individual functioning levels in the process of stroke diagnosis and treatment.

## Data Availability Statement

The datasets presented in this article are readily available. Requests to access the datasets should be directed to the corresponding author.

## Ethics Statement

The studies involving human participants were reviewed and approved by the Ethics Committee of The First Rehabilitation Hospital of Shanghai (IRB# YK-2021-02-011) and The Affiliated Sir Run Run Hospital of Nanjing Medical University (IRB# 2018-SR-017). The patients/participants provided their written informed consent to participate in this study.

## Author Contributions

CF designed the protocol, recruited subjects, and wrote the manuscript. FL contributed to the research concept, supervised the entire study, proofread the manuscript, performed the analysis, and generated the images. ZLJ contributed to the research concept and proofread the manuscript. MXS helped recruit the subjects and gave useful suggestions. All authors contributed to the article and approved the submitted version.

## Conflict of Interest

The authors declare that the research was conducted in the absence of any commercial or financial relationships that could be construed as a potential conflict of interest.

## Publisher's Note

All claims expressed in this article are solely those of the authors and do not necessarily represent those of their affiliated organizations, or those of the publisher, the editors and the reviewers. Any product that may be evaluated in this article, or claim that may be made by its manufacturer, is not guaranteed or endorsed by the publisher.
