# Supplementary material for: Simplified Post-stroke Functioning Assessment Based on ICF via Dichotomous Mokken Scale Analysis and Rasch Modeling
Source: Front Neurol. 2022 Apr 14;13:827247. doi: 10.3389/fneur.2022.827247 (PMC9046681; doi:10.3389/fneur.2022.827247)
Supplement: Supplementary file 1 [file Table_1.docx]

Appendix 1. The body function (b) and activity and participation (d) aspects of the Extended Comprehensive ICF Core Set for Stroke.

Items are listed with their code, title, and whether they were selected (denoted as T) for Mokken analysis or not (denoted as F). The dropped items were with ≥ 5% missing data in the sample population.

| code | category title | selected |  | code | category title | selected |  |  |
| --- | --- | --- | --- | --- | --- | --- | --- | --- |
| b110 | Consciousness functions | T |  | d120 | Other purposeful sensing | T |  |  |
| b114 | Orientation functions | T |  | d130 | Copying | T |  |  |
| b117 | Intellectual functions | T |  | d135 | Rehearsing | T |  |  |
| b126 | Temperament and personality functions | T |  | d155 | Acquiring skills | T |  |  |
| b130 | Energy and drive functions | T |  | d160 | Focusing attention | T |  |  |
| b134 | Sleep functions | T |  | d172 | Calculating | T |  |  |
| b140 | Attention functions | T |  | d175 | Solving problems | T |  |  |
| b144 | Memory functions | T |  | d177 | Making decisions | T |  |  |
| b147 | Psychomotor functions | T |  | d210 | Undertaking a single task | T |  |  |
| b152 | Emotional functions | T |  | d220 | Undertaking multiple tasks | T |  |  |
| b156 | Perceptual functions | T |  | d230 | Carrying out daily routine | T |  |  |
| b160 | Thought functions | T |  | d240 | Handling stress and other psychological demands | T |  |  |
| b164 | Higher-level cognitive functions | T |  | d310 | Communicating with - receiving - spoken messages | T |  |  |
| b167 | Mental functions of language | T |  | d315 | Communicating with - receiving - nonverbal messages | T |  |  |
| b172 | Calculation functions | T |  | d330 | Speaking | T |  |  |
| b176 | Mental function of sequencing complex movements | T |  | d335 | Producing nonverbal messages | T |  |  |
| b180 | Experience of self and time functions | T |  | d350 | Conversation | T |  |  |
| b210 | Seeing functions | T |  | d410 | Changing basic body position | T |  |  |
| b215 | Functions of structures adjoining the eye | T |  | d415 | Maintaining a body position | T |  |  |
| b230 | Hearing functions | T |  | d420 | Transferring oneself | T |  |  |
| b235 | Vestibular functions | T |  | d430 | Lifting and carrying objects | T |  |  |
| b240 | Sensations associated with hearing and vestibular function | T |  | d440 | Fine hand use | T |  |  |
| b260 | Proprioceptive function | T |  | d445 | Hand and arm use | T |  |  |
| b265 | Touch function | T |  | d450 | Walking | T |  |  |
| b270 | Sensory functions related to temperature and other stimuli | T |  | d510 | Washing oneself | T |  |  |
| b280 | Sensation of pain | T |  | d520 | Caring for body parts | T |  |  |
| b310 | Voice functions | T |  | d530 | Toileting | T |  |  |
| b320 | Articulation functions | T |  | d540 | Dressing | T |  |  |
| b330 | Fluency and rhythm of speech functions | T |  | d550 | Eating | T |  |  |
| b340 | Alternative vocalization functions | T |  | d560 | Drinking | T |  |  |
| b410 | Heart functions | T |  | d570 | Looking after one's health | T |  |  |
| b415 | Blood vessel functions | T |  | d710 | Basic interpersonal interactions | T |  |  |
| b420 | Blood pressure functions | T |  | d760 | Family relationships | T |  |  |
| b430 | Haematological system functions | T |  | d770 | Intimate relationships | T |  |  |
| b435 | Immunological system functions | T |  | b640 | Sexual functions | F |  |  |
| b440 | Respiration functions | T |  | b770 | Gait pattern functions | F |  |  |
| b450 | Additional respiratory functions | T |  | d166 | Reading | F |  |  |
| b455 | Exercise tolerance functions | T |  | d170 | Writing | F |  |  |
| b510 | Ingestion functions | T |  | d325 | Communicating with - receiving - written messages | F |  |  |
| b515 | Digestive functions | T |  | d345 | Writing messages | F |  |  |
| b525 | Defecation functions | T |  | d360 | Using communication devices and techniques | F |  |  |
| b530 | Weight maintenance functions | T |  | d455 | Moving around | F |  |  |
| b535 | Sensations associated with the digestive system | T |  | d460 | Moving around in different locations | F |  |  |
| b540 | General metabolic functions | T |  | d465 | Moving around using equipment | F |  |  |
| b545 | Water, mineral and electrolyte balance functions | T |  | d470 | Using transportation | F |  |  |
| b550 | Thermoregulatory functions | T |  | d475 | Driving | F |  |  |
| b620 | Urination functions | T |  | d620 | Acquisition of goods and services | F |  |  |
| b630 | Sensations associated with urinary functions | T |  | d630 | Preparing meals | F |  |  |
| b710 | Mobility of joint functions | T |  | d640 | Doing housework | F |  |  |
| b715 | Stability of joint functions | T |  | d750 | Informal social relationships | F |  |  |
| b730 | Muscle power functions | T |  | d845 | Acquiring, keeping, and terminating a job | F |  |  |
| b735 | Muscle tone functions | T |  | d850 | Remunerative employment | F |  |  |
| b740 | Muscle endurance functions | T |  | d855 | Non-remunerative employment | F |  |  |
| b750 | Motor reflex functions | T |  | d860 | Basic economic transactions | F |  |  |
| b755 | Involuntary movement reaction functions | T |  | d870 | Economic self-sufficiency | F |  |  |
| b760 | Control of voluntary movement functions | T |  | d910 | Community life | F |  |  |
| b810 | Protective functions of the skin | T |  | d920 | Recreation and leisure | F |  |  |
| d110 | Watching | T |  | d930 | Religion and spirituality | F |  |  |
| d115 | Listening | T |  | d940 | Human rights | F |  |  |
