# Supplementary material for: Simplified Post-stroke Functioning Assessment Based on ICF via Dichotomous Mokken Scale Analysis and Rasch Modeling
Source: Front Neurol. 2022 Apr 14;13:827247. doi: 10.3389/fneur.2022.827247 (PMC9046681; doi:10.3389/fneur.2022.827247)
Supplement: Supplementary file 2 [file Table_2.docx]

Appendix 2. Outcome patterns of AISP with different lower bound of scalability coefficients.

| lower bound | max scale | unscalable items (%) | items in scale 1 (%) |
| --- | --- | --- | --- |
| 0.30 | 5 | 26.88 | 62.37 |
| 0.33 | 4 | 23.66 | 65.59 |
| 0.36 | 3 | 31.18 | 59.14 |
| 0.39 | 2 | 37.63 | 58.06 |
| ***0.42*** | ***4*** | ***37.63*** | ***53.76*** |
| 0.45 | 3 | 44.09 | 48.39 |
| ***0.48*** | ***3*** | ***46.24*** | ***45.16*** |
| 0.51 | 2 | 51.61 | 38.71 |
| 0.54 | 3 | 50.54 | 41.94 |
