# Supplementary material for: Simplified Post-stroke Functioning Assessment Based on ICF via Dichotomous Mokken Scale Analysis and Rasch Modeling
Source: Front Neurol. 2022 Apr 14;13:827247. doi: 10.3389/fneur.2022.827247 (PMC9046681; doi:10.3389/fneur.2022.827247)
Supplement: Supplementary file 3 [file Table_3.docx]

Appendix 3. Outcome of AISP with 0.42 as cut value of scalability coefficient. The items in scale 0 are unscalable.

| Scale | Code | Category Title | Scale | Code | Category Title |
| --- | --- | --- | --- | --- | --- |
| 0 | b134 | Sleep functions | 1 | b110 | Consciousness functions |
| 0 | b147 | Psychomotor functions | 1 | b117 | Intellectual functions |
| 0 | b156 | Perceptual functions | 1 | b126 | Temperament and personality functions |
| 0 | b210 | Seeing functions | 1 | b130 | Energy and drive functions (G) |
| 0 | b215 | Functions of structures adjoining the eye | 1 | b140 | Attention functions |
| 0 | b230 | Hearing functions | 1 | b160 | Thought functions |
| 0 | b235 | Vestibular functions | 1 | b164 | Higher-level cognitive functions |
| 0 | b240 | Sensations associated with hearing and vestibular function | 1 | b167 | Mental functions of language |
| 0 | b260 | Proprioceptive function | 1 | b172 | Calculation functions |
| 0 | b265 | Touch function | 1 | b176 | Mental function of sequencing complex movements |
| 0 | b270 | Sensory functions related to temperature and other stimuli | 1 | b180 | Experience of self and time functions |
| 0 | b280 | Sensation of pain (G) | 1 | b310 | Voice functions |
| 0 | b340 | Alternative vocalization functions | 1 | b320 | Articulation functions |
| 0 | b410 | Heart functions | 1 | b330 | Fluency and rhythm of speech functions |
| 0 | b420 | Blood pressure functions | 1 | b430 | Haematological system functions |
| 0 | b435 | Immunological system functions | 1 | b450 | Additional respiratory functions |
| 0 | b440 | Respiration functions | 1 | b455 | Exercise tolerance functions |
| 0 | b510 | Ingestion functions | 1 | b540 | General metabolic functions |
| 0 | b515 | Digestive functions | 1 | b550 | Thermoregulatory functions |
| 0 | b525 | Defecation functions | 1 | b730 | Muscle power functions |
| 0 | b535 | Sensations associated with the digestive system | 1 | b740 | Muscle endurance functions |
| 0 | b620 | Urination functions | 1 | b755 | Involuntary movement reaction functions |
| 0 | b630 | Sensations associated with urinary functions | 1 | b760 | Control of voluntary movement functions |
| 0 | b710 | Mobility of joint functions | 1 | d120 | Other purposeful sensing |
| 0 | b715 | Stability of joint functions | 1 | d130 | Copying |
| 0 | b735 | Muscle tone functions | 1 | d135 | Rehearsing |
| 0 | b750 | Motor reflex functions | 1 | d160 | Focusing attention |
| 0 | d110 | Watching | 1 | d175 | Solving problems |
| 0 | d115 | Listening | 1 | d177 | Making decisions |
| 0 | d155 | Acquiring skills | 1 | d210 | Undertaking a single task |
| 0 | d172 | Calculating | 1 | d220 | Undertaking multiple tasks |
| 0 | d240 | Handling stress and other psychological demands | 1 | d230 | Carrying out daily routine (G) |
| 0 | d415 | Maintaining a body position | 1 | d310 | Communicating with - receiving - spoken messages |
| 0 | d760 | Family relationships | 1 | d315 | Communicating with - receiving - nonverbal messages |
| 0 | d770 | Intimate relationships | 1 | d330 | Speaking |
| 2 | b114 | Orientation functions | 1 | d335 | Producing nonverbal messages |
| 2 | b144 | Memory functions | 1 | d350 | Conversation |
| 2 | b810 | Protective functions of the skin | 1 | d410 | Changing basic body position |
| 3 | b415 | Blood vessel functions | 1 | d420 | Transferring oneself |
| 3 | b530 | Weight maintenance functions | 1 | d440 | Fine hand use |
| 3 | b545 | Water, mineral and electrolyte balance functions | 1 | d445 | Hand and arm use |
| 4 | b152 | Emotional functions (G) | 1 | d450 | Walking (G) |
| 4 | d430 | Lifting and carrying objects | 1 | d510 | Washing oneself |
|  |  |  | 1 | d520 | Caring for body parts |
|  |  |  | 1 | d530 | Toileting |
|  |  |  | 1 | d540 | Dressing |
|  |  |  | 1 | d550 | Eating |
|  |  |  | 1 | d560 | Drinking |
|  |  |  | 1 | d570 | Looking after one's health |
|  |  |  | 1 | d710 | Basic interpersonal interactions |
