# Supplementary material for: Simplified Post-stroke Functioning Assessment Based on ICF via Dichotomous Mokken Scale Analysis and Rasch Modeling
Source: Front Neurol. 2022 Apr 14;13:827247. doi: 10.3389/fneur.2022.827247 (PMC9046681; doi:10.3389/fneur.2022.827247)
Supplement: Supplementary file 4 [file Table_4.docx]

Appendix 4. Homogeneity coefficients for the 50 items in scale 1 from AISP. H_i_ is the homogeneity/scalability coefficient of a single item. The overall coefficient of the whole set was also listed at the bottom line of table.

| code | ICF Category Title | H_i_ | SE |
| --- | --- | --- | --- |
| ***b110*** | ***Consciousness functions*** | ***0.6692*** | ***0.1325*** |
| ***b117*** | ***Intellectual functions*** | ***0.7138*** | ***0.1086*** |
| b126 | Temperament and personality functions | 0.5792 | 0.0731 |
| b130 | Energy and drive functions (G) | 0.4877 | 0.0734 |
| b140 | Attention functions | 0.4883 | 0.0689 |
| b160 | Thought functions | 0.5482 | 0.0855 |
| b164 | Higher-level cognitive functions | 0.5381 | 0.0669 |
| b167 | Mental functions of language | 0.4469 | 0.0991 |
| b172 | Calculation functions | 0.4422 | 0.0694 |
| b176 | Mental function of sequencing complex movements | 0.5331 | 0.0724 |
| ***b180*** | ***Experience of self and time functions*** | ***0.5290*** | ***0.1017*** |
| b310 | Voice functions | 0.4779 | 0.0803 |
| b320 | Articulation functions | 0.4852 | 0.0798 |
| b330 | Fluency and rhythm of speech functions | 0.4215 | 0.0748 |
| ***b430*** | ***Haematological system functions*** | ***0.4281*** | ***0.1578*** |
| ***b450*** | ***Additional respiratory functions*** | ***0.4451*** | ***0.1159*** |
| b455 | Exercise tolerance functions | 0.4803 | 0.0580 |
| ***b540*** | ***General metabolic functions*** | ***0.7310*** | ***0.1964*** |
| b550 | Thermoregulatory functions | 0.9704 | 0.0009 |
| b730 | Muscle power functions | 0.5537 | 0.0644 |
| b740 | Muscle endurance functions | 0.4911 | 0.0651 |
| b755 | Involuntary movement reaction functions | 0.4501 | 0.0675 |
| b760 | Control of voluntary movement functions | 0.5398 | 0.0581 |
| d120 | Other purposeful sensing | 0.4362 | 0.0901 |
| d130 | Copying | 0.5529 | 0.0622 |
| d135 | Rehearsing | 0.5609 | 0.0838 |
| d160 | Focusing attention | 0.6043 | 0.0541 |
| d175 | Solving problems | 0.5909 | 0.0542 |
| d177 | Making decisions | 0.5948 | 0.0643 |
| d210 | Undertaking a single task | 0.6143 | 0.0538 |
| d220 | Undertaking multiple tasks | 0.4666 | 0.0620 |
| d230 | Carrying out daily routine (G) | 0.5807 | 0.0534 |
| d310 | Communicating with - receiving - spoken messages | 0.6256 | 0.0943 |
| d315 | Communicating with - receiving - nonverbal messages | 0.5806 | 0.0843 |
| d330 | Speaking | 0.5680 | 0.0686 |
| d335 | Producing nonverbal messages | 0.5342 | 0.0885 |
| d350 | Conversation | 0.5287 | 0.0703 |
| d410 | Changing basic body position | 0.5716 | 0.0535 |
| d420 | Transferring oneself | 0.6108 | 0.0470 |
| d440 | Fine hand use | 0.4967 | 0.0851 |
| d445 | Hand and arm use | 0.5820 | 0.0610 |
| d450 | Walking (G) | 0.6411 | 0.0466 |
| d510 | Washing oneself | 0.7413 | 0.0441 |
| d520 | Caring for body parts | 0.6817 | 0.0430 |
| d530 | Toileting | 0.5707 | 0.0515 |
| d540 | Dressing | 0.6364 | 0.0418 |
| d550 | Eating | 0.4938 | 0.0652 |
| d560 | Drinking | 0.5031 | 0.0663 |
| d570 | Looking after one's health | 0.6068 | 0.0489 |
| d710 | Basic interpersonal interactions | 0.5723 | 0.0551 |
| Overall | H | 0.5446 | 0.0437 |
