# Supplementary material for: Simplified Post-stroke Functioning Assessment Based on ICF via Dichotomous Mokken Scale Analysis and Rasch Modeling
Source: Front Neurol. 2022 Apr 14;13:827247. doi: 10.3389/fneur.2022.827247 (PMC9046681; doi:10.3389/fneur.2022.827247)
Supplement: Supplementary file 5 [file Table_5.docx]

Appendix 5. Monotonicity checking for the 50 items in scale 1 from AISP.

#ac: the number of possible violations; #vi: the actual number of violations; #zsig: number of significant violations; Crit: a critical value summarizes the effect size of violation.

| code | #ac | #vi | #zsig | Crit |  | code | #ac | #vi | #zsig | Crit |
| --- | --- | --- | --- | --- | --- | --- | --- | --- | --- | --- |
| b110 | 1 | 0 | 0 | 0 |  | d135 | 1 | 0 | 0 | 0 |
| b117 | 1 | 0 | 0 | 0 |  | d160 | 1 | 0 | 0 | 0 |
| b126 | 1 | 0 | 0 | 0 |  | d175 | 1 | 0 | 0 | 0 |
| b130 | 1 | 0 | 0 | 0 |  | d177 | 1 | 0 | 0 | 0 |
| b140 | 1 | 0 | 0 | 0 |  | d210 | 1 | 0 | 0 | 0 |
| b160 | 1 | 0 | 0 | 0 |  | d220 | 1 | 0 | 0 | 0 |
| b164 | 1 | 0 | 0 | 0 |  | d230 | 1 | 0 | 0 | 0 |
| b167 | 1 | 0 | 0 | 0 |  | d310 | 1 | 0 | 0 | 0 |
| b172 | 1 | 0 | 0 | 0 |  | d315 | 0 | 0 | 0 | 0 |
| b176 | 1 | 0 | 0 | 0 |  | d330 | 1 | 0 | 0 | 0 |
| b180 | 1 | 0 | 0 | 0 |  | d335 | 1 | 0 | 0 | 0 |
| b310 | 1 | 0 | 0 | 0 |  | d350 | 1 | 0 | 0 | 0 |
| b320 | 1 | 0 | 0 | 0 |  | d410 | 1 | 0 | 0 | 0 |
| b330 | 1 | 0 | 0 | 0 |  | d420 | 1 | 0 | 0 | 0 |
| b430 | 1 | 0 | 0 | 0 |  | d440 | 1 | 0 | 0 | 0 |
| b450 | 1 | 0 | 0 | 0 |  | d445 | 1 | 0 | 0 | 0 |
| b455 | 1 | 0 | 0 | 0 |  | d450 | 1 | 0 | 0 | 0 |
| b540 | 0 | 0 | 0 | 0 |  | d510 | 0 | 0 | 0 | 0 |
| b550 | 0 | 0 | 0 | 0 |  | d520 | 0 | 0 | 0 | 0 |
| b730 | 1 | 0 | 0 | 0 |  | d530 | 1 | 0 | 0 | 0 |
| b740 | 1 | 0 | 0 | 0 |  | d540 | 1 | 0 | 0 | 0 |
| b755 | 1 | 0 | 0 | 0 |  | d550 | 1 | 0 | 0 | 0 |
| b760 | 1 | 0 | 0 | 0 |  | d560 | 1 | 0 | 0 | 0 |
| d120 | 1 | 0 | 0 | 0 |  | d570 | 1 | 0 | 0 | 0 |
| d130 | 1 | 0 | 0 | 0 |  | d710 | 1 | 0 | 0 | 0 |
