# Supplementary material for: Simplified Post-stroke Functioning Assessment Based on ICF via Dichotomous Mokken Scale Analysis and Rasch Modeling
Source: Front Neurol. 2022 Apr 14;13:827247. doi: 10.3389/fneur.2022.827247 (PMC9046681; doi:10.3389/fneur.2022.827247)
Supplement: Supplementary file 6 [file Table_6.docx]

Appendix 6. Invariant Item Ordering checking for the 50 items in scale 1 from AISP.

#ac: the number of possible violations; #vi: the actual number of violations; #zsig: number of significant violations; Crit: a critical value summarizes the extent of violation. Bold italic items were the ones deleted by the backward item selection procedure.

| code | #ac | #vi | #zsig | Crit |  | code | #ac | #vi | #zsig | Crit |
| --- | --- | --- | --- | --- | --- | --- | --- | --- | --- | --- |
| b110 | 49 | 0 | 0 | 0 |  | d135 | 49 | 1 | 0 | 2 |
| b117 | 49 | 0 | 0 | 0 |  | d160 | 49 | 9 | 1 | 52 |
| b126 | 49 | 1 | 0 | 4 |  | d175 | 49 | 2 | 0 | 3 |
| b130 | 49 | 2 | 0 | 14 |  | d177 | 49 | 2 | 0 | 7 |
| b140 | 49 | 2 | 0 | 11 |  | d210 | 49 | 6 | 0 | 29 |
| b160 | 49 | 0 | 0 | 0 |  | d220 | 49 | 4 | 0 | 23 |
| b164 | 49 | 0 | 0 | 0 |  | d230 | 49 | 1 | 0 | -3 |
| ***b167*** | ***49*** | ***8*** | ***1*** | ***52*** |  | d310 | 49 | 0 | 0 | 0 |
| b172 | 49 | 1 | 0 | 11 |  | d315 | 49 | 0 | 0 | 0 |
| b176 | 49 | 2 | 0 | 10 |  | d330 | 49 | 2 | 0 | 5 |
| b180 | 49 | 0 | 0 | 0 |  | d335 | 49 | 0 | 0 | 0 |
| b310 | 49 | 4 | 0 | 20 |  | d350 | 49 | 1 | 0 | 3 |
| b320 | 49 | 0 | 0 | 0 |  | d410 | 49 | 1 | 0 | -2 |
| ***b330*** | ***49*** | ***5*** | ***1*** | ***50*** |  | d420 | 49 | 2 | 0 | 8 |
| b430 | 49 | 0 | 0 | 0 |  | d440 | 49 | 0 | 0 | 0 |
| b450 | 49 | 0 | 0 | 0 |  | d445 | 49 | 0 | 0 | 0 |
| b455 | 49 | 0 | 0 | 0 |  | d450 | 49 | 0 | 0 | 0 |
| b540 | 49 | 0 | 0 | 0 |  | d510 | 49 | 0 | 0 | 0 |
| b550 | 49 | 0 | 0 | 0 |  | d520 | 49 | 0 | 0 | 0 |
| b730 | 49 | 0 | 0 | 0 |  | d530 | 49 | 0 | 0 | 0 |
| b740 | 49 | 1 | 0 | 3 |  | d540 | 49 | 1 | 0 | -4 |
| ***b755*** | ***49*** | ***4*** | ***1*** | ***47*** |  | d550 | 49 | 1 | 0 | 9 |
| b760 | 49 | 2 | 0 | 7 |  | d560 | 49 | 2 | 0 | 10 |
| d120 | 49 | 1 | 0 | 5 |  | d570 | 49 | 2 | 1 | 33 |
| d130 | 49 | 2 | 1 | 24 |  | d710 | 49 | 0 | 0 | 0 |
