# Supplementary material for: Simplified Post-stroke Functioning Assessment Based on ICF via Dichotomous Mokken Scale Analysis and Rasch Modeling
Source: Front Neurol. 2022 Apr 14;13:827247. doi: 10.3389/fneur.2022.827247 (PMC9046681; doi:10.3389/fneur.2022.827247)
Supplement: Supplementary file 7 [file Table_7.docx]

Appendix 7**. Item difficulties of the final Rasch model composed of 45 ICF categories.**

SE: standard error. The *p* values were corrected by Bonferroni method.

| code | category title | difficulty | SE | z score | χ^2^ | *p* | *adj.p* |
| --- | --- | --- | --- | --- | --- | --- | --- |
| d510 | Washing oneself | 2.2249 | 0.2758 | 8.0677 | 27.0168 | 0.0014 | 0.0625 |
| d520 | Caring for body parts | 1.6819 | 0.2507 | 6.7084 | 19.1273 | 0.0241 | 1 |
| d450 | Walking | 1.5748 | 0.247 | 6.3752 | 16.5358 | 0.0565 | 1 |
| b730 | Muscle power functions | 1.126 | 0.2354 | 4.7842 | 12.1678 | 0.204 | 1 |
| d445 | Hand and arm use | 1.126 | 0.2354 | 4.7842 | 10.2271 | 0.3324 | 1 |
| b740 | Muscle endurance functions | 0.8936 | 0.2315 | 3.8595 | 6.97 | 0.6402 | 1 |
| d540 | Dressing | 0.6234 | 0.2288 | 2.7248 | 18.0952 | 0.0341 | 1 |
| b455 | Exercise tolerance functions | 0.4022 | 0.2278 | 1.7656 | 7.1435 | 0.6222 | 1 |
| d530 | Toileting | 0.3583 | 0.2277 | 1.5735 | 9.5895 | 0.3847 | 1 |
| d410 | Changing basic body position | 0.0491 | 0.2284 | 0.2148 | 10.2508 | 0.3306 | 1 |
| d220 | Undertaking multiple tasks | -0.0842 | 0.2294 | -0.3669 | 10.2549 | 0.3302 | 1 |
| d420 | Transferring oneself | -0.2199 | 0.2307 | -0.9531 | 19.0345 | 0.0249 | 1 |
| b760 | Control of voluntary movement functions | -0.311 | 0.2318 | -1.3415 | 9.1217 | 0.4261 | 1 |
| d570 | Looking after one's health | -0.4031 | 0.2331 | -1.7293 | 10.434 | 0.3165 | 1 |
| d230 | Carrying out daily routine | -0.8381 | 0.2415 | -3.47 | 9.8509 | 0.3627 | 1 |
| d710 | Basic interpersonal interactions | -1.0446 | 0.2469 | -4.2308 | 4.7354 | 0.8567 | 1 |
| d175 | Solving problems | -1.2068 | 0.2518 | -4.7937 | 10.7818 | 0.291 | 1 |
| d550 | Eating | -1.2628 | 0.2536 | -4.9802 | 5.7463 | 0.765 | 1 |
| d160 | Focusing attention | -1.3199 | 0.2555 | -5.1667 | 9.3659 | 0.4042 | 1 |
| d560 | Drinking | -1.4965 | 0.2618 | -5.7155 | 6.563 | 0.6825 | 1 |
| b140 | Attention functions | -1.5569 | 0.2642 | -5.8937 | 5.1743 | 0.8189 | 1 |
| d210 | Undertaking a single task | -1.8148 | 0.2751 | -6.5975 | 10.116 | 0.3412 | 1 |
| b130 | Energy and drive functions | -1.8149 | 0.2751 | -6.5975 | 11.5205 | 0.2417 | 1 |
| b176 | Mental function of sequencing complex movements | -1.8831 | 0.2782 | -6.7681 | 12.2237 | 0.201 | 1 |
| b164 | Higher-level cognitive functions | -1.9528 | 0.2816 | -6.9352 | 4.4835 | 0.8768 | 1 |
| d350 | Conversation | -2.0243 | 0.2851 | -7.0996 | 7.0803 | 0.6288 | 1 |
| d177 | Making decisions | -2.1749 | 0.293 | -7.4219 | 17.5664 | 0.0406 | 1 |
| d130 | Copying | -2.254 | 0.2974 | -7.5782 | 4.3651 | 0.8858 | 1 |
| b310 | Voice functions | -2.254 | 0.2974 | -7.5782 | 5.8515 | 0.7547 | 1 |
| d330 | Speaking | -2.254 | 0.2974 | -7.5782 | 8.829 | 0.4532 | 1 |
| b320 | Articulation functions | -2.4194 | 0.3072 | -7.8757 | 5.4219 | 0.7961 | 1 |
| d135 | Rehearsing | -2.5064 | 0.3127 | -8.0167 | 11.5704 | 0.2386 | 1 |
| b126 | Temperament and personality functions | -2.5064 | 0.3127 | -8.0167 | 9.338 | 0.4067 | 1 |
| d335 | Producing nonverbal messages | -2.6926 | 0.3251 | -8.282 | 14.2092 | 0.1151 | 1 |
| b160 | Thought functions | -2.6926 | 0.3251 | -8.282 | 25.3277 | 0.0026 | 0.1183 |
| d120 | Other purposeful sensing | -2.6926 | 0.3251 | -8.282 | 10.2646 | 0.3295 | 1 |
| b180 | Experience of self and time functions | -2.895 | 0.34 | -8.5154 | 11.7012 | 0.2307 | 1 |
| d315 | Communicating with - receiving - nonverbal messages | -3.1203 | 0.3583 | -8.7093 | 2.7263 | 0.9742 | 1 |
| d310 | Communicating with - receiving - spoken messages | -3.2442 | 0.3692 | -8.7872 | 6.4262 | 0.6966 | 1 |
| b450 | Additional respiratory functions | -3.515 | 0.3954 | -8.8887 | 7.4167 | 0.5938 | 1 |
| b117 | Intellectual functions | -3.6681 | 0.4119 | -8.9063 | 8.6257 | 0.4725 | 1 |
| b110 | Consciousness functions | -4.0217 | 0.4547 | -8.8444 | 7.6989 | 0.5648 | 1 |
| b430 | Haematological system functions | -4.2287 | 0.4834 | -8.7469 | 8.7225 | 0.4633 | 1 |
| b540 | General metabolic functions | -5.1022 | 0.6438 | -7.9254 | 4.8963 | 0.8433 | 1 |
| b550 | Thermoregulatory functions | -5.5853 | 0.7694 | -7.2595 | 2.7444 | 0.9736 | 1 |
