# Supplementary material for: Simplified Post-stroke Functioning Assessment Based on ICF via Dichotomous Mokken Scale Analysis and Rasch Modeling
Source: Front Neurol. 2022 Apr 14;13:827247. doi: 10.3389/fneur.2022.827247 (PMC9046681; doi:10.3389/fneur.2022.827247)
Supplement: Supplementary file 8 [file Table_8.docx]

Appendix 8. Lord’s χ^2^ test for differential item functioning analysis of gender.

The Lord’s χ^2^ statistics are listed in the table. The *p* values were adjusted by the Holm method. DIF effect-size values are measured via the delta Lord (ΔLord). The degree of effect-size can be classified by the absolute values of ΔLord. ΔLord <1 is the negligible effect. 1.5>ΔLord ≥1.0 is the moderate effect. ΔLord≥1.5 is the large effect. The items were firstly tested for DIF or non-DIF. Then each DIF item (with Holm adjusted *p* value < 0.05) was classified by its effect-size.

| code | Lord’s χ^2^ | *p* | Holm *adj.p* | ΔLord |  | code | Lord’s χ2 | p | Holm *adj.p* | ΔLord |
| --- | --- | --- | --- | --- | --- | --- | --- | --- | --- | --- |
| b110 | 0.347 | 0.5558 | 0.8933 | 1.6967 |  | d175 | 0.1788 | 0.6724 | 0.9621 | -0.5711 |
| b117 | 0.6871 | 0.4072 | 0.8328 | -1.7277 |  | d177 | 0.8509 | 0.3563 | 0.7635 | -1.4079 |
| b126 | 0.103 | 0.7483 | 0.9621 | -0.5372 |  | d210 | 1.6717 | 0.196 | 0.5189 | -1.8408 |
| b130 | 0.0259 | 0.8722 | 0.9799 | -0.2397 |  | d220 | 1.9227 | 0.1656 | 0.4967 | -1.6932 |
| b140 | 1.5428 | 0.2142 | 0.5355 | -1.7094 |  | d230 | 2.2263 | 0.1357 | 0.4697 | -1.8948 |
| b160 | 1.9258 | 0.1652 | 0.4967 | 3.0031 |  | d310 | 0.0244 | 0.8759 | 0.9799 | -0.3111 |
| b164 | 0.0182 | 0.8928 | 0.9799 | 0.2092 |  | d315 | 0.0015 | 0.9693 | 0.9984 | 0.0759 |
| b176 | 6.5377 | 0.0106 | 0.1584 | -3.5774 |  | d330 | 0.6176 | 0.4319 | 0.8451 | 1.3672 |
| b180 | 0.1135 | 0.7362 | 0.9621 | -0.6105 |  | d335 | 0.4522 | 0.5013 | 0.8676 | -1.1428 |
| b310 | 1.1859 | 0.2762 | 0.654 | -1.6730 |  | d350 | 0.3471 | 0.5558 | 0.8933 | 0.9579 |
| b320 | 0.0242 | 0.8763 | 0.9799 | -0.2590 |  | d410 | 7.8046 | 0.0052 | 0.1173 | -3.4216 |
| b430 | 0.484 | 0.4866 | 0.8676 | -1.6918 |  | d420 | 3.0312 | 0.0817 | 0.3679 | -2.1314 |
| b450 | 0.0754 | 0.7836 | 0.9795 | 0.6199 |  | d445 | 3.0496 | 0.0808 | 0.3679 | -2.2191 |
| b455 | 5.2978 | 0.0214 | 0.2402 | -2.8235 |  | d450 | 3.9718 | 0.0463 | 0.347 | -2.7185 |
| b540 | 0.2734 | 0.601 | 0.9327 | -1.6817 |  | d510 | 0.9377 | 0.3329 | 0.749 | -1.4100 |
| b550 | 0 | 0.9994 | 0.9994 | 46.7455 |  | d520 | 3.0297 | 0.0818 | 0.3679 | -2.3848 |
| b730 | 4.7828 | 0.0287 | 0.2587 | -2.8200 |  | d530 | 2.3934 | 0.1219 | 0.4697 | -1.8826 |
| b740 | 3.6047 | 0.0576 | 0.3679 | -2.3740 |  | d540 | 0.1645 | 0.6851 | 0.9621 | -0.4940 |
| b760 | 2.3388 | 0.1262 | 0.4697 | -1.8805 |  | d550 | 0.0014 | 0.9702 | 0.9984 | -0.0515 |
| d120 | 0.5106 | 0.4749 | 0.8676 | 1.3719 |  | d560 | 0.0009 | 0.9762 | 0.9984 | -0.0425 |
| **d130** | **11.8378** | **0.0006** | **0.0261** | **-5.0603** |  | d570 | 1.7247 | 0.1891 | 0.5189 | -1.6246 |
| d135 | 0.1029 | 0.7483 | 0.9621 | -0.5370 |  | d710 | 0.2102 | 0.6466 | 0.9621 | 0.6272 |
| d160 | 0.0276 | 0.868 | 0.9799 | -0.2298 |  |  |  |  |  |  |
