# Supplementary material for: Simplified Post-stroke Functioning Assessment Based on ICF via Dichotomous Mokken Scale Analysis and Rasch Modeling
Source: Front Neurol. 2022 Apr 14;13:827247. doi: 10.3389/fneur.2022.827247 (PMC9046681; doi:10.3389/fneur.2022.827247)
Supplement: Supplementary file 9 [file Data_Sheet_1.PDF]

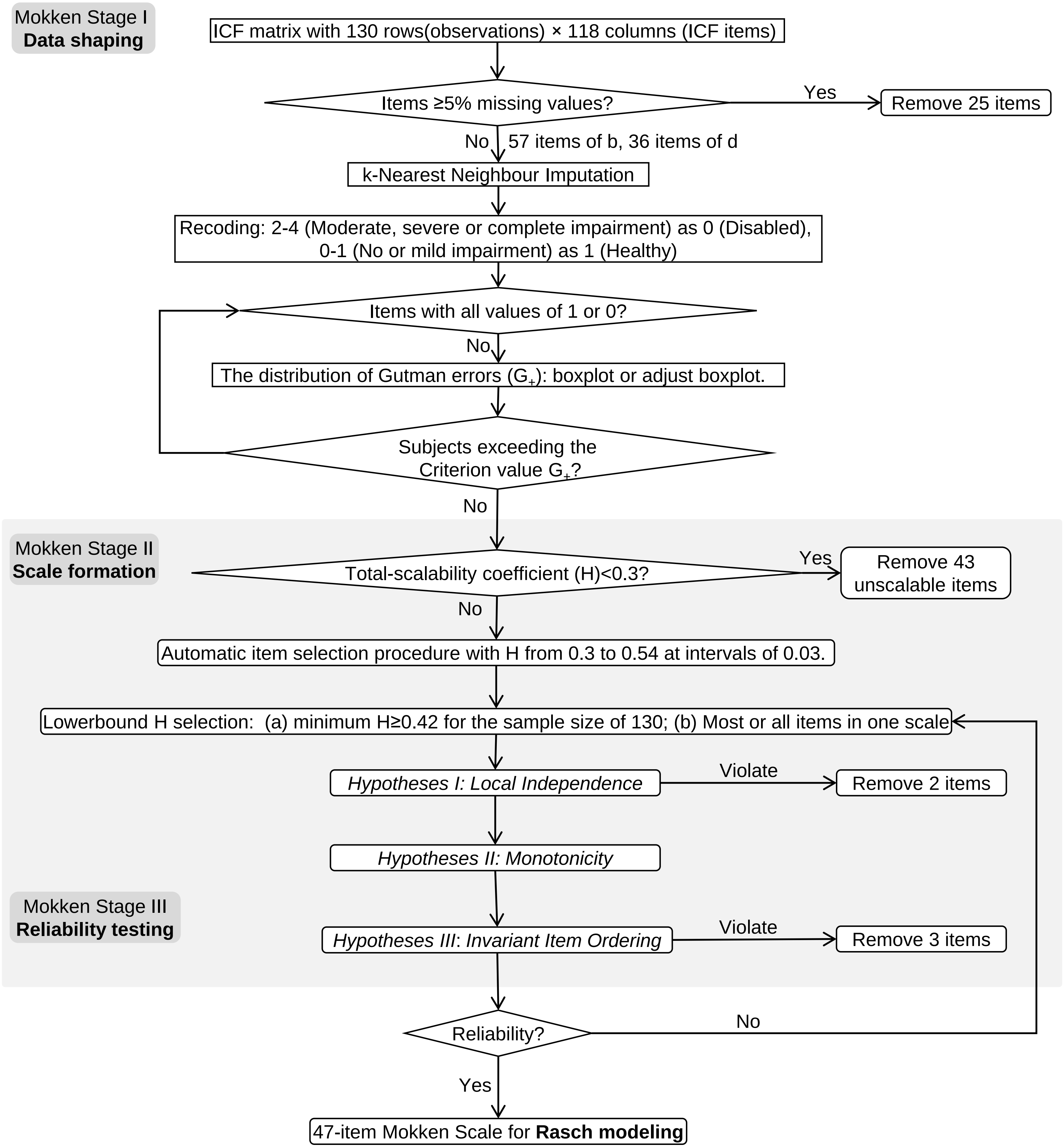

Mokken Stage I
Data shaping
ICF matrix with 130 rows(observations) × 118 columns (ICF items)
Items ≥5% missing values?
Yes
Remove 25 items
No
57 items of b, 36 items of d
k-Nearest Neighbour Imputation
Recoding: 2-4 (Moderate, severe or complete impairment) as 0 (Disabled), 0-1 (No or mild impairment) as 1 (Healthy)
Items with all values of 1 or 0?
No
The distribution of Gutman errors (G_+): boxplot or adjust boxplot.
Subjects exceeding the Criterion value G_+?
No
Mokken Stage II
Scale formation
Total-scalability coefficient (H)<0.3?
Yes
Remove 43 unscalable items
No
Automatic item selection procedure with H from 0.3 to 0.54 at intervals of 0.03.
Lowerbound H selection: (a) minimum H≥0.42 for the sample size of 130; (b) Most or all items in one scale
Hypotheses I: Local Independence
Violate
Remove 2 items
Hypotheses II: Monotonicity
Mokken Stage III
Reliability testing
Hypotheses III: Invariant Item Ordering
Violate
Remove 3 items
Reliability?
No
Yes
47-item Mokken Scale for Rasch modeling
