# Supplementary material for: Simplified Post-stroke Functioning Assessment Based on ICF via Dichotomous Mokken Scale Analysis and Rasch Modeling
Source: Front Neurol. 2022 Apr 14;13:827247. doi: 10.3389/fneur.2022.827247 (PMC9046681; doi:10.3389/fneur.2022.827247)
Supplement: Supplementary file 10 [file Data_Sheet_2.PDF]

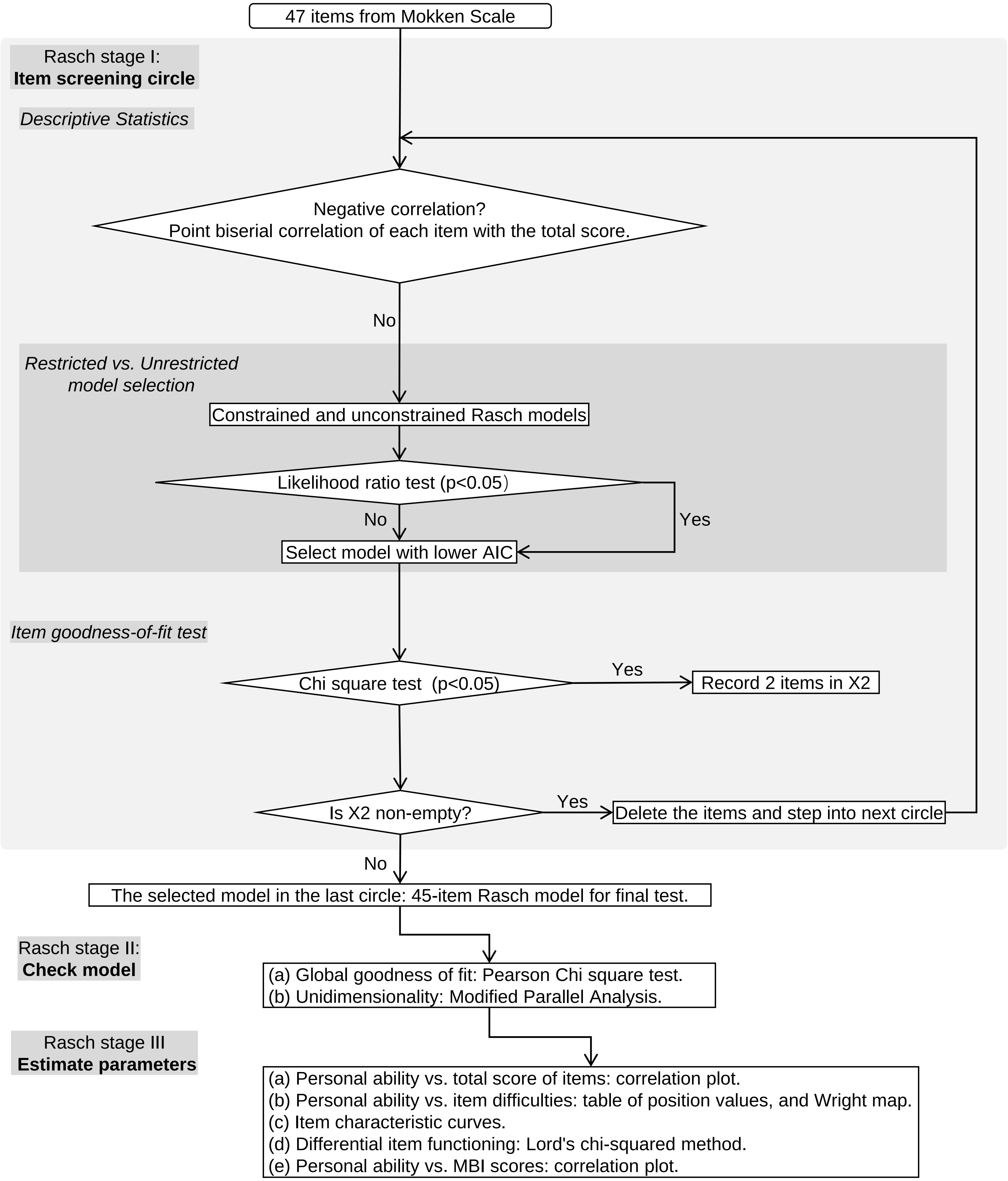
47 items from Mokken Scale
Rasch stage I:
Item screening circle
Descriptive Statistics
Negative correlation?
Point biserial correlation of each item with the total score.
No
Restricted vs. Unrestricted
model selection
Constrained and unconstrained Rasch models
Likelihood ratio test (p<0.05)
No
Yes
Select model with lower AIC
Item goodness-of-fit test
Chi square test (p<0.05)
Yes
Record 2 items in X2
Is X2 non-empty?
Yes
Delete the items and step into next circle
No
The selected model in the last circle: 45-item Rasch model for final test.
Rasch stage II:
Check model
(a) Global goodness of fit: Pearson Chi square test.
(b) Unidimensionality: Modified Parallel Analysis.
Rasch stage III
Estimate parameters
(a) Personal ability vs. total score of items: correlation plot.
(b) Personal ability vs. item difficulties: table of position values, and Wright map.
(c) Item characteristic curves.
(d) Differential item functioning: Lord's chi-squared method.
(e) Personal ability vs. MBI scores: correlation plot.
